# Historical tree phenology data reveal the seasonal rhythms of the Congo Basin rainforest

**DOI:** 10.1002/pei3.10136

**Published:** 2024-03-11

**Authors:** Elizabeth Kearsley, Hans Verbeeck, Piet Stoffelen, Steven B. Janssens, Emmanuel Kasongo Yakusu, Margaret Kosmala, Tom De Mil, Marijn Bauters, Elasi Ramanzani Kitima, José Mbifo Ndiapo, Adelard Lonema Chuda, Andrew D. Richardson, Lisa Wingate, Bhély Angoboy Ilondea, Hans Beeckman, Jan van den Bulcke, Pascal Boeckx, Koen Hufkens

**Affiliations:** ^1^ Computational and Applied Vegetation Ecology Lab, Department of Environment, Faculty of Bioscience Engineering Ghent University Gent Belgium; ^2^ BlueGreen Labs Melsele Belgium; ^3^ Meise Botanic Garden Meise Belgium; ^4^ Department of Biology, Leuven Plant Institute KULeuven Leuven Belgium; ^5^ UGent‐Woodlab (Laboratory of Wood Technology), Department of Environment, Faculty of Bioscience Engineering Ghent University Gent Belgium; ^6^ Service of Wood Biology Royal Museum for Central Africa Tervuren Belgium; ^7^ Faculté de gestion des ressources naturelles renouvelables Université de Kisangani Kisangani Democratic Republic of Congo; ^8^ Department of Organismic and Evolutionary Biology Harvard University Cambridge Massachusetts USA; ^9^ CIBO Technologies Cambridge Massachusetts USA; ^10^ Forest is Life, TERRA Teaching and Research Centre, Gembloux Agro Bio‐Tech University of Liège Gembloux Belgium; ^11^ Isotope Bioscience Laboratory ‐ ISOFYS, Department of Green Chemistry and Technology Ghent University Gent Belgium; ^12^ Research Group of Plants and Ecosystems (PLECO), Department of Biology University of Antwerp Wilrijk Belgium; ^13^ Institut National pour l'Etude et la Recherche Agronomiques‐INERA Yangambi Democratic Republic of Congo; ^14^ Center for Ecosystem Science and Society Northern Arizona University Flagstaff Arizona USA; ^15^ School of Informatics, Computing and Cyber Systems Northern Arizona University Flagstaff Arizona USA; ^16^ INRAE, UMR ISPA Villenave d'Ornon France; ^17^ Institut National pour l'Étude et la Recherche Agronomiques Kinshasa Democratic Republic of Congo

**Keywords:** leaf senescence, leaf turnover, phenology, scaling, tropical forest

## Abstract

Tropical forest phenology directly affects regional carbon cycles, but the relation between species‐specific and whole‐canopy phenology remains largely uncharacterized. We present a unique analysis of historical tropical tree phenology collected in the central Congo Basin, before large‐scale impacts of human‐induced climate change. Ground‐based long‐term (1937–1956) phenological observations of 140 tropical tree species are recovered, species‐specific phenological patterns analyzed and related to historical meteorological records, and scaled to characterize stand‐level canopy dynamics. High phenological variability within and across species and in climate–phenology relationships is observed. The onset of leaf phenophases in deciduous species was triggered by drought and light availability for a subset of species and showed a species‐specific decoupling in time along a bi‐modal seasonality. The majority of the species remain evergreen, although central African forests experience relatively low rainfall. Annually a maximum of 1.5% of the canopy is in leaf senescence or leaf turnover, with overall phenological variability dominated by a few deciduous species, while substantial variability is attributed to asynchronous events of large and/or abundant trees. Our results underscore the importance of accounting for constituent signals in canopy‐wide scaling and the interpretation of remotely sensed phenology signals.

## Graphical abstract

Ground‐based long‐term (1937–1956) phenological observations of tropical tree species in the central Congo Basin are recovered and allow for the analysis of species‐specific phenological patterns. The onset of leaf phenophases in deciduous species was triggered by drought and light availability for a subset of species and showed a species‐specific decoupling in time along the bi‐modal seasonality. Few deciduous species dominate stand‐level canopy dynamics while substantial variability is attributed to asynchronous events of large and/or abundant trees.

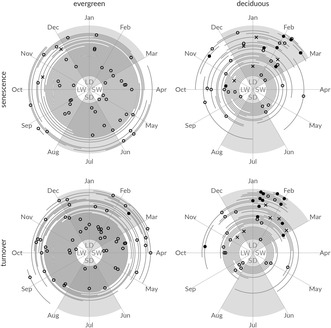

## 1 INTRODUCTION

Plant phenological events are regulated by environmental drivers including temperature, water, and light availability to varying degrees (Bradley et al., 2011; Schwartz et al., 2006; Wright & van Schaik, 1994). Variability in prevailing climate can therefore cause shifts in the timing of key plant phenophases such as leaf appearance and senescence as well as flowering and fruiting (Butt et al., 2015; Richardson et al., 2013; Schwartz et al., 2006). The first‐order control of vegetation activity at the biosphere–atmosphere interface actively contributes to the modification of climate, all the way from local to global scales (Parmesan & Yohe, 2003; Peñuelas et al., 2009). As phenology affects the timing of critical ecosystem processes such as the dynamics of carbon, water and energy fluxes, nutrient cycling, and canopy conductance (Richardson et al., 2013), it is increasingly important to understand how variations in climate have affected ecosystem function in the past to predict with more certainty how ecosystems will respond in the future.

Presently, relationships describing the interactions between climate and phenology in tropical forests are limited (Abernethy et al., 2018; Richardson et al., 2013; Wright & Calderón, 2018), despite these forests playing a significant role in the Earth's carbon budget and strongly influencing regional and global climate (Lewis, 2006; Pan et al., 2011). Typically, vegetation seasonality in tropical forests is assessed via synoptic observations derived from litterfall monitoring (reviewed by Chave et al., 2010) or optical remote sensing data (Bradley et al., 2011; Wu et al., 2016). From this, important patterns underlying variability in tropical leaf phenology have been derived and mainly converge on the finding that when seasonally varying precipitation is not limiting, light availability is a major driver for tropical forest phenology (Bradley et al., 2011; Chave et al., 2010; Guan et al., 2015; Philippon et al., 2016). Furthermore, satellite analyses have suggested that the tropical evergreen state is not sustained year‐round if mean annual precipitation is roughly below 2000 mm (Guan et al., 2015). This seems specifically important for the Congo Basin, which stands out pan‐tropically for being drier than the Amazon Basin or South‐East Asia (Feng et al., 2013; Guan et al., 2015), and exhibiting bi‐modal rather than unimodal seasonality (Knoben et al., 2019). For the central African “moist” forest belt, however, we have little process‐based understanding of the forest's phenological patterns. Furthermore, it could be argued that (medium resolution) remote sensing alone is inappropriate for assessing phenology, particularly when species diversity is high. In this context, previous studies have shown that in the absence of major driving forces, species, and even individuals seem to display a wide range of phenological behavior (Reich, 1995; van Schaik et al., 1993) and are potentially responding to different cues. Thus, a more complete characterization of the periodicity, synchrony, and drivers of leaf phenology at the individual and species level is imperative to understand tropical tree phenology at the ecosystem scale and to predict how stand‐level phenology may alter with changing climate. Nevertheless, few long‐term high‐frequency phenology measurements are made of tropical tree species (e.g., Bush et al., 2018; Wright & Calderón, 2018) as the challenges posed by such large species diversity (Slik et al., 2015) and the sustained monitoring work involved over many years to decades are by no means trivial. Even less data traces back to periods preceding the climate change disturbances experienced today (Lewis & Maslin, 2015), providing sparse baseline measurements of tree phenology.

Here we present a scaling analysis on a historical long‐term observational data set (1937–1956) of tropical rainforest tree phenology at the Yangambi research station situated in the center of the Congo Basin. We use quasi‐weekly observations of leaf senescence and turnover of 140 species (668 trees, 5011 individual observation years), from a larger phenology database, representing 96.0% of the historical stand‐level basal area of this forest (Pierlot, 1966; Table S1). We discuss this unique dense data set and the inter‐ and intraspecies seasonality within the context of a stand‐level scaling exercise, using historical basal area measurements. An analysis of cross‐correlation to climate variables and Fourier‐based (cyclical) seasonality analysis is used to understand a canopy‐wide phenology and the constituent signals of our canopy‐wide scaling. We argue for contemporary ground‐based long‐term monitoring with high coverage of species in tropical forests to understand both historical and current phenological behavior and how phenology in these multi‐species ecosystems scales to the landscape level.

## 2 METHODOLOGY

### 2.1 Study area

Historical data were recovered from a phenological study carried out between 1937 and 1956 in the central Congo Basin at the Yangambi Research Station (N 00°48′, E 24°29′) in what is now the Democratic Republic of Congo (DRC). The data were retrieved from the archives of the INERA (Institut National pour l'Etude et la Recherche Agronomique) herbarium at the Yangambi Research Station. Historically, phenological observations in the Congo Basin were made as part of a wider research effort that was initiated by the “Institut National pour l'Étude Agronomique du Congo” (INEAC) to describe the phenology of two different forests from the late 1930s to the late 1950s (Coppieters, 2013; Couralet et al., 2013).

The Yangambi forest reserve (now a UNESCO Man and Biosphere reserve) is situated ~100 km west of the city of Kisangani, in the central Congo Basin, with an average elevation of 459 masl. The largest part of the reserve consists of semi‐deciduous old‐growth forest. The central Congo Basin rainforests experience a weak seasonal climate with a bi‐modal periodicity of wet and dry seasons (Malhi & Wright, 2004) generated by the movement of the Intertropical Convergence Zone. The reserve receives an annual precipitation of 1842 ± 254 mm (in situ meteorological data, 1931–2012) with two dry seasons with monthly precipitation lower than 150 mm, during December–February and June–July (95% confidence interval on the long‐term mean). Average temperatures are high and constant throughout the year with a minimum of 24.2 ± 0.4°C in July and a maximum of 25.5 ± 0.6°C in March. The geology of the region consists of unconsolidated eolian sedimentary sandy material of Pleistocene age, giving way to Xanthic Ferralsols (sensu van Engelen et al., 2006; WRB 2014).

### 2.2 Historical data recovery

Ground‐based phenological observations of local tropical trees were made four times each month on a rotating schedule (resolution of 7.2 days on average) from 1937 until 1956 by the forestry division of the INEAC (Institut National pour l’Étude Agronomique du Congo). The sampling protocol was recovered at the State Archives of Belgium, including details on the observation methods, the observational routes, and the training and schedules of the observers. Canopy leaf senescence was defined as a distinct period during which leaves fall and trees remain bare, while canopy turnover was defined as a period during which leaf fall comes in peaks with concomitant flushes of new leaves (INEAC archives). Summarized data sheets of these long‐term observations were digitized using 12 MP resolution cameras. The hand‐written notes and annotations depicting phenophases during the observational period were digitized to binary data (yes or no phenophase event at each time‐step) through an online citizen science project “Jungle Rhythms” (Hufkens & Kearsley, 2023, https://www.zooniverse.org/projects/khufkens/jungle‐rhythms). The complete recovered phenological data set represents 2073 individual trees and 13,373 individual observation years covering 635 species and 115 genus‐level identifications (total of 388 genera and 78 families), and 57 unidentified individuals. In addition to the leaf phenological phenophases, events of flowering, fruiting, and seed dispersal were also recorded.

Quality control and validation of the data produced through citizen science were performed using an independent validation dataset made by the authors for ~2% of the data, where all phenophases were directly transcribed into text‐format data indicating the exact weeks of the observation. Overall, our classification results were highly accurate with overall accuracies using Kappa values, ranging from a low value of 0.56 up to 0.97, with values between 0.81 and 1 considered to be in almost perfect agreement. All outliers detected during data analysis were visually checked in the digitized full data sheet (e.g., to control for dust specks on the original copy).

Meteorological data were recovered from the archives of the climatological station in Yangambi. Data from precipitation were available starting in 1931, while temperature and sun hours were added in 1951, with measurements ongoing.

Historical forest inventory data of dense evergreen to semi‐deciduous forests of the Yangambi reserve have been retrieved. Inventory data was collected by the forestry division of INEAC Yangambi, and compiled by Pierlot (1966). These mixed forests were characterized by the presence of *Scorodophloeus zenkeri* Harms and *Cola griseiflora* De Wild, a dominant forest type of the reserve. Three systematic surveys were carried out of large forest areas, in which bands of 10 m wide and 1 km long were inventoried: (1) Inventory 20: Systematic survey of a 1200 ha block, at 3% survey intensity, a total of 40 ha; (2) Inventory 21: Systematic survey of a 100 ha block, at 10% survey intensity, a total of 10 ha; and (3) Inventory 23: Systematic survey of a stand (unknown full area), with a total of 12 ha inventoried. All trees with a minimum circumference of 20 cm were measured at 1.50 m from the ground or above the buttress, and identified to species level. All data was compiled at species level in circumference categories of 20 by 20 cm, providing the number of individuals per category.

### 2.3 Species selection

In our phenological analysis, we use a selection of species found in the historical forest inventories (Pierlot, 1966), representing 96.0% of the basal area within these inventories. The final phenological data used comprises 668 individuals covering 140 species (representing 112 genera and 38 families) for a total of 5011 individual observation years (overview of species in Table S1). We did not include individuals that were only identified to the genus level.

Descriptions of leafing phenology (evergreen, deciduous) of all species were compiled via a literature search of published floras (Arbonnier, 2004; Hawthorne & Jongkind, 2006; Lemmens et al., 2012; Meerts & Hasson, 2016; Meunier et al., 2015). For 29 species no classification was found in literature, and for 8 species leaf phenology in literature was described as sometimes deciduous depending on the environment. When the phenological data from this study provided a clear deciduous/evergreen classification for these species, an a‐posteriori classification was assigned (Table S1). A total of 8 species remain unclassified. The leaf phenology classifications are used to structure the results but are not part of the leaf phenological data analysis.

### 2.4 Data analysis

Species‐specific phenological patterns are assessed across the entire observational period as the average of the investigated individuals. The annual patterns are the average across individuals and all observed years. Species‐specific annual median timing of the onset of the phenophases, that is, the first week of the observed phenophase, was assessed using circular statistics (R package “circular,” Agostinelli & Lund, 2017) to allow for the connection of December to January. The degree of phenological variability across years (interannual variability) and individuals (intraspecies variability), that is, the degree of timing divergence, was assessed as the circular standard deviation in timing of the onset of each phenophase within and across individuals, respectively (following Wang et al., 2016). In addition, a Fourier analysis is performed to detect potential patterns in the species‐level phenological time series (R package “stats,” function “spectrum,” R Core Team, 2020). The method detailed in Bush et al. (2017) has been applied, in which dominant cycles are identified from smoothed spectral estimates and compared to a null continuum (by applying the Daniell kernel to provide a smooth periodogram with the bandwidth of approximately 1) to determine the confidence. Dominant cycles with a 95% confidence are retained.

Species‐specific cross‐correlation analysis between the time series of the phenological observations and climatological variables was performed at a monthly resolution (R package “tseries,” function ccf, Trapletti & Hornik, 2019) to identify in‐phase or lags of the two time series. Separate cross‐correlations between the climatic predictor variables precipitation, sun hours and maximum temperature, and response variables canopy turnover and senescence are assessed. This analysis was performed for species with a minimum of five observed phenophases (across individuals) in the time series and significant correlations of *p* < .05 were retained. For the time series of precipitation, the in situ monthly data from 1936 to 1956 was used. For the variables “sun hours” and “maximum temperature,” only limited overlap in these time series was available (1951–1956) and cross‐correlations were assessed using a repetitive time series of averaged monthly data from these years. Time series were a priori tested for trend stationarity using the ADF (Augmented Dickey‐Fuller Test; Fuller, 1996) and KPSS (Hobijn et al., 2004) tests and for auto‐correlation (R package “forecast,” function “acf,” Hyndman et al., 2020). To account for the auto‐correlation within the climate time series as a bi‐modal system (auto‐correlation of −.236 at 3 months; *p* < .05), the investigated lags were limited to 3 months.

Aggregated phenological patterns at the stand level were assessed by weighing the annual species‐specific patterns according to their basal area at the stand level. Basal area is selected to represent a species’ proportion of the canopy as it is shown to explain a significant level of crown area variability pan‐tropically, with limited variation in scaling parameters across sites (Blanchard et al., 2016). Species groups characterized by the cross‐correlation and Fourier‐based seasonality analysis were assessed separately for their contribution to the stand‐level phenological signal, with their contribution to this signal represented by the area under the curve. Climate‐correlated classes are assigned through k‐means clustering of the correlation coefficients (*K* = 3, number of clusters selected through minimizing within groups sum of squares). Seasonality‐related classes are assigned as annual and nonannual. Here, species with a subannual pattern are included within the annual class as the phenophase will occur on multiple occasions within a year.

### 2.5 Comparison with EVI dynamics

Mean annual dynamics of EVI for old‐growth forest locations in Yangambi (Kearsley et al., 2013) were computed using the Terra MODerate Resolution Imaging Spectroradiometer (MODIS) Vegetation Indices (MOD13Q1) data from 2000 to 2018 (Didan, 2015). The EVI signal is solely used to assess the general agreement in ground‐based observational phenological patterns and a remote sensing‐based signal. Potential ongoing shifts in phenology due to changes in climate or species composition are not accounted for. The 60+ year mismatch in timing should be noted.

## 3 RESULTS

**FIGURE 1:**
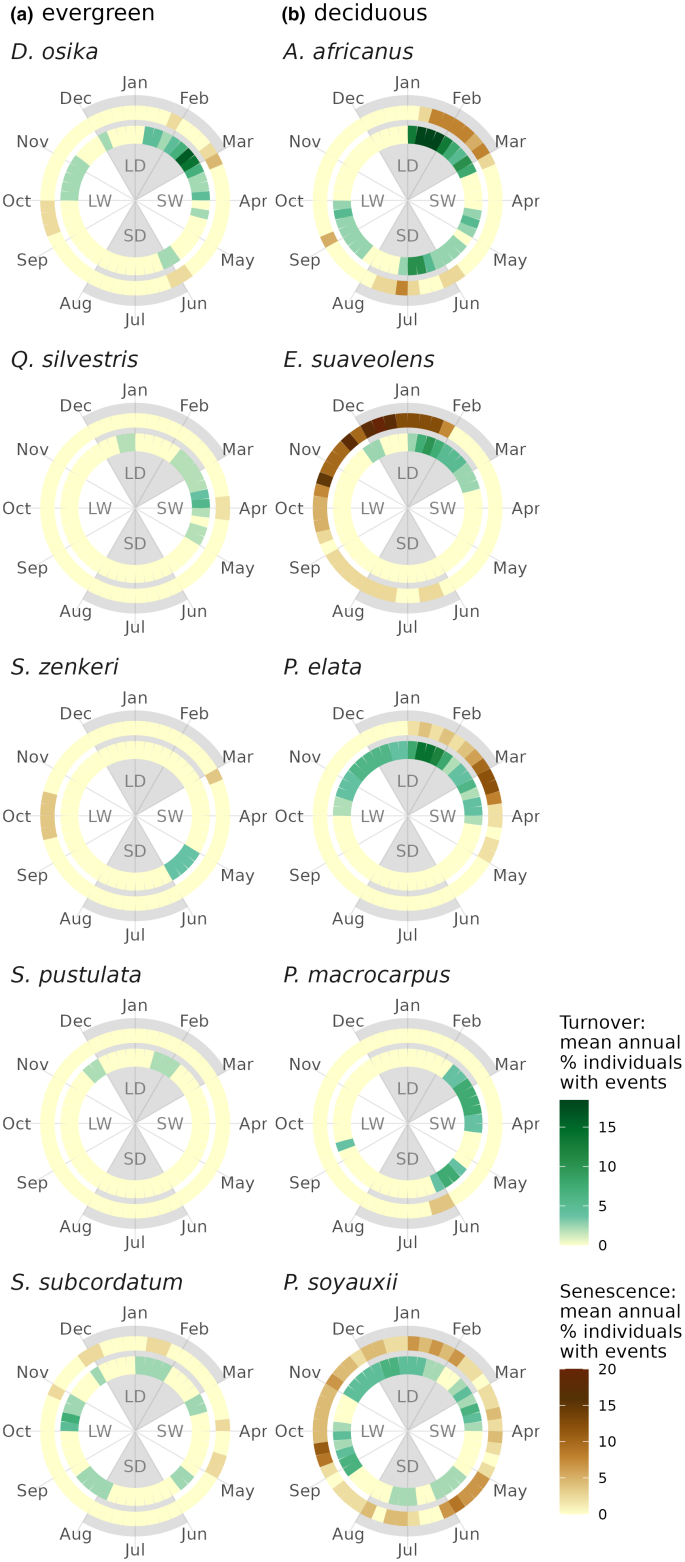
Overview of leaf phenological cycles for a set of exemplary (a) evergreen and (b) deciduous species. The average annual pattern for each species with the percentage of individuals with events indicated using a color scale. Leaf senescence is indicated in the outer circle in brown, turnover in the inner circle in green. Gray areas indicate the average timing of the long and short dry seasons (LD and SD; monthly precipitation <150 mm), separated by the long and short wet seasons (LW and SW). The leaf phenological patterns for all 140 species can be found in Figure S1.

High heterogeneity in species‐specific leaf phenology was found within this equatorial tropical forest, irrespective of their prior classifications in the literature as evergreen (*N* = 89) or deciduous (*N* = 43). This is demonstrated by species‐specific variation in the total number of phenophase occurrences (i.e., in which a tree canopy is in a state of phenological event) during the observational period, the timing and duration of phenophases, as well as the synchrony of this timing across years and individual trees (e.g., species see Figure 1; Table 1; all species in Figure S1; Tables S2 and S3). The majority of evergreen species (~71%; by literature review) exhibited leaf phenological activity perceptible for an observer from the ground across the observation period. These were generally events of canopy turnover mostly only occurring sporadically, followed by lengthy periods of time without leaf phenological activity (i.e., several years or even decades) (e.g., *S. zenkeri* and *Strombosia pustulata*). For a few evergreens, sporadic canopy‐wide senescence was also observed (e.g., *Dacryodes osika* and *S. zenkeri*). The phenological patterns found within the species classified as evergreen, although highly variable, were significantly distinguishable from the species classified as deciduous. The deciduous tree species showed overall higher occurrences of leaf phenophases (*p* < .001), fewer years without observed leaf phenophases (*p* < .001), and a higher synchrony in the timing of these phenophases across the years. Observed patterns (Table 1; Figure 1) varied from annual cycles with low interannual variability (e.g., senescence at the beginning or end of the long dry season for *Erythrophleum suaveolens* and *Pericopsis elata*, respectively), to multiple intraannual cycles (e.g., *Allophyllus africanus* with leaf turnover every 6 months), or seemingly random (e.g., *Pterocarpus soyauxii*).

**TABLE 1:** Species‐specific characteristics of the phenological cycles of leaf senescence and turnover for a set of example species.

Species	nr ind	nr obs years	% years with phase	Duration phase (w)	Variability (SD)		Cyclicity	Cross‐correlations		
					intra‐sp.	inter‐ann.		precip	Sun hours	tmax
Leaf senescence										
*Dacryodes osika*	4	43	11.6	2 ± 0.9	11.4					
*Quassia silvestris*	6	52	1.9	2.2						
*Scorodophloeus zenkeri*	4	27	11.1	1.8 ± 1.3	11.4					
*Strombosia pustulata*	5	48	0.0							
*Synsepalum subcordatum*	8	41	9.8	1.7 ± 0.6	10.9	9.3				
*Allophylus africanus*	7	38	34.2	2 ± 0.6	12.3	8.4 ± 7.3		−0.155 (t0)	0.172 (t0)	0.172 (t0)
*Erythrophleum suaveolens*	5	40	50.0	4 ± 2.5	6.7	5.9 ± 2.2	Annual	0.197 (t‐2)	−0.194 (t‐1)	−0.188 (t0)
*Pericopsis elata*	5	50	40.0	2 ± 0.8	3.5	1.8 ± 0.6	Annual	−0.128 (t0)	0.26 (t0)	0.347 (t0)
*Petersianthus macrocarpus*	3	26	3.8	2.2						
*Pterocarpus soyauxii*	6	45	75.6	2 ± 1.4	20.2	14.1 ± 1.9				
Leaf turnover										
*D. osika*	4	43	25.6	3 ± 1.8	7.6	6.3 ± 2.7	Annual		0.161 (t0)	0.245 (t0)
*Q. silvestris*	6	52	5.8	3.2 ± 3.1	6.2	4.1			0.144 (t‐1)	0.158 (t0)
*S. zenkeri*	4	27	3.7	4.3						
*S. pustulata*	5	48	4.2	2.7 ± 0.8						
*S. subcordatum*	8	41	14.6	2.7 ± 1.2	12.6	10.4				
*A. africanus*	7	38	50.0	3.4 ± 1.7	10.5	8.5 ± 2.1	Subannual	−0.215 (t0)	0.235 (t0)	0.228 (t0)
*E. suaveolens*	5	40	20.0	3 ± 0.8	3.4	3.4	Annual	−0.263 (t0)	0.32 (t0)	0.259 (t0)
*P. elata*	5	50	34.0	4.2 ± 3.5	7	4 ± 1.9	Annual	−0.133 (t0)	0.196 (t0)	−0.129 (t‐2)
*P. macrocarpus*	3	26	19.2	3.4 ± 1.4	10.1	10.1	Subannual	−0.159 (t‐1)	0.192 (t‐1)	0.219 (t0)
*P. soyauxii*	6	45	40.0	2.7 ± 1.7	14.4	11.6 ± 2.9			−0.155 (t‐3)	

*Note*: Five evergreen (top) and five deciduous (bottom) species are presented, matching those in Figure 1. For each species, the number of individuals observed, the total observation years, the percentage of observation years with observed phenophases, and the average duration (±SD) of a phenophase in weeks (w) are indicated. Variability in the timing of phenophases across years is represented as interannual SD and within species (across individuals) as intraspecies SD. Timing of Fourier‐based cyclicity is indicated. Significant correlations of time‐series cross‐correlation analysis between phenological observations and precipitation, amount of sun hours, and maximum temperature are provided, with time of lag of the phenological observations in relation to each climatological variable indicated. Time t0 indicates significant in‐phase cross‐correlations (*p* < .05); t‐x indicates the time lag in months of significantly lagged cross‐correlations (*p* < .05).

**FIGURE 2:**
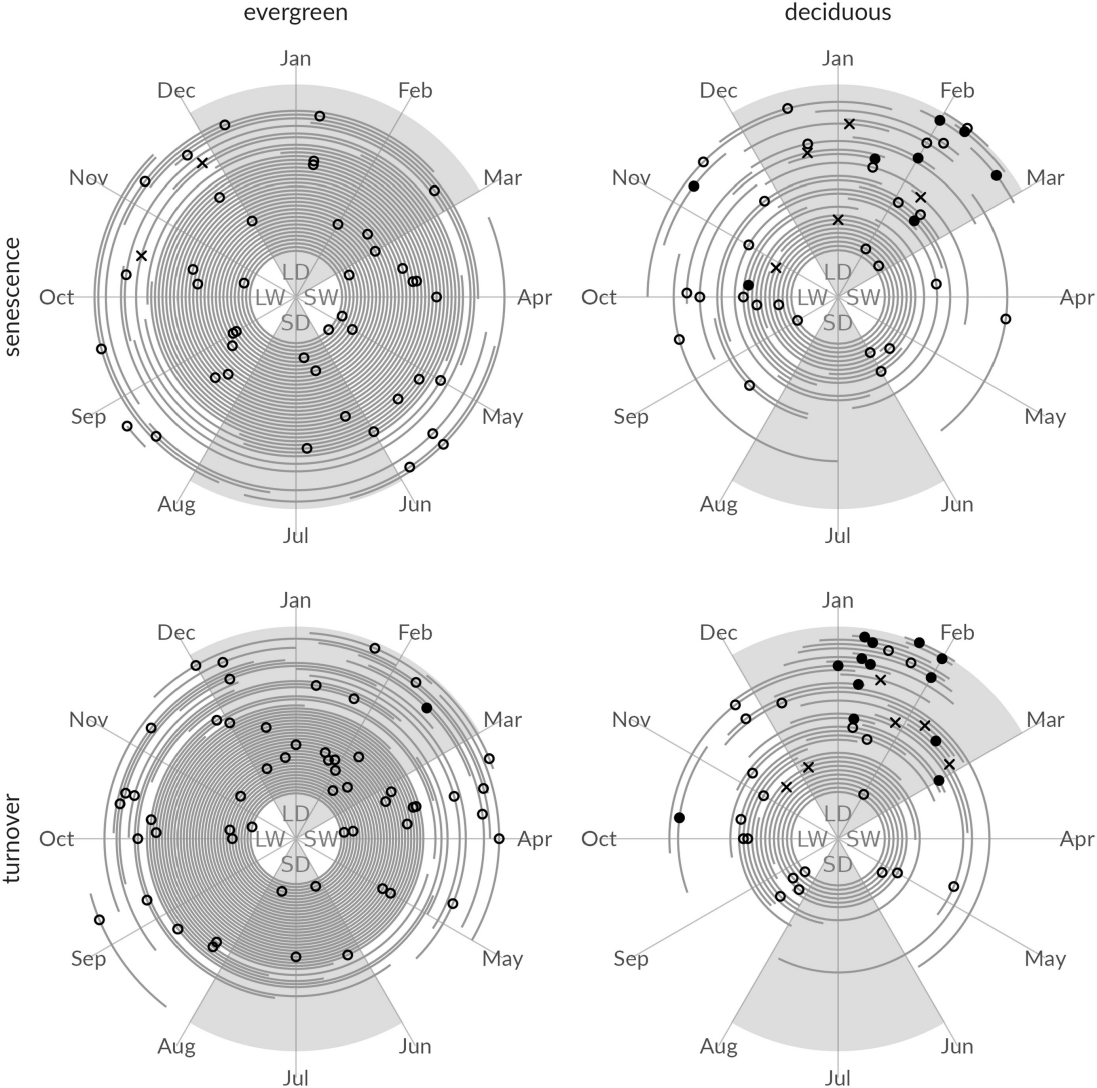
Overview of species‐specific timing of onset of phenophases for evergreen and deciduous species. The median timing of the onset of leaf senescence and turnover is indicated for each species. Species‐specific bootstrapped 95% confidence intervals are indicated with a line segment. Species are arranged according to the variability in the timing of the phenophase, with species with the lowest variability at the outer edge and continuing toward the center. The symbol used for timing of onset distinguishes species with an annual (full circles) or sub‐annual (crosses) Fourier‐based seasonality and those without annual seasonality (open circles). Gray‐shaded areas represent the average timing of the long and short dry seasons (LD and SD; monthly precipitation <150 mm), separated by the long and short wet seasons (LW and SW).

The large variety of leaf phenological patterns, or lack of patterns, across all species, translates into year‐round divergence in the timing of these phenophases across an average year. The species‐specific median timing of the onset of both senescence and turnover shows that canopy leaf events can be initiated at any point in time over the year (Figure 2).

Several deciduous species show synchrony in the timing of onset of events (Figure 2, right panels) with a group of species for which both senescence and turnover are initiated during the long dry season (Dec–Feb), dominated by annual seasonal patterns (as determined using Fourier‐based analysis). The interannual and intraspecies variability of these deciduous species with phenophase onset during the long dry season was significantly lower (*p* < .001; 4.8 ± 2.5 weeks and 7.1 ± 3.7 weeks, respectively) compared to deciduous species with phenophase onsets during the rest of the year (9.6 ± 3.6 weeks and 11.4 ± 4.4 weeks, respectively).

Evergreen species diverge in their timing of phenophases (Figure 2, left panels). Due to the low level of phenophase recurrences over the years, a high uncertainty in the timing of events (confidence intervals extended year‐round) is present. Evergreens with higher numbers of phenophase recurrences show similar values for interannual and intraspecies variability as those of deciduous species (7.8 ± 3.9 weeks and 9.9 ± 4.4 weeks, respectively), although no apparent interspecies synchrony is visible. Overall, phenophases were short in duration, with an average canopy senescence period lasting 2.4 ± 1.3 weeks and average canopy turnover taking place in 4.0 ± 4.2 weeks, which did not differ significantly between evergreen or deciduous species (*p* > .1).

**FIGURE 3:**
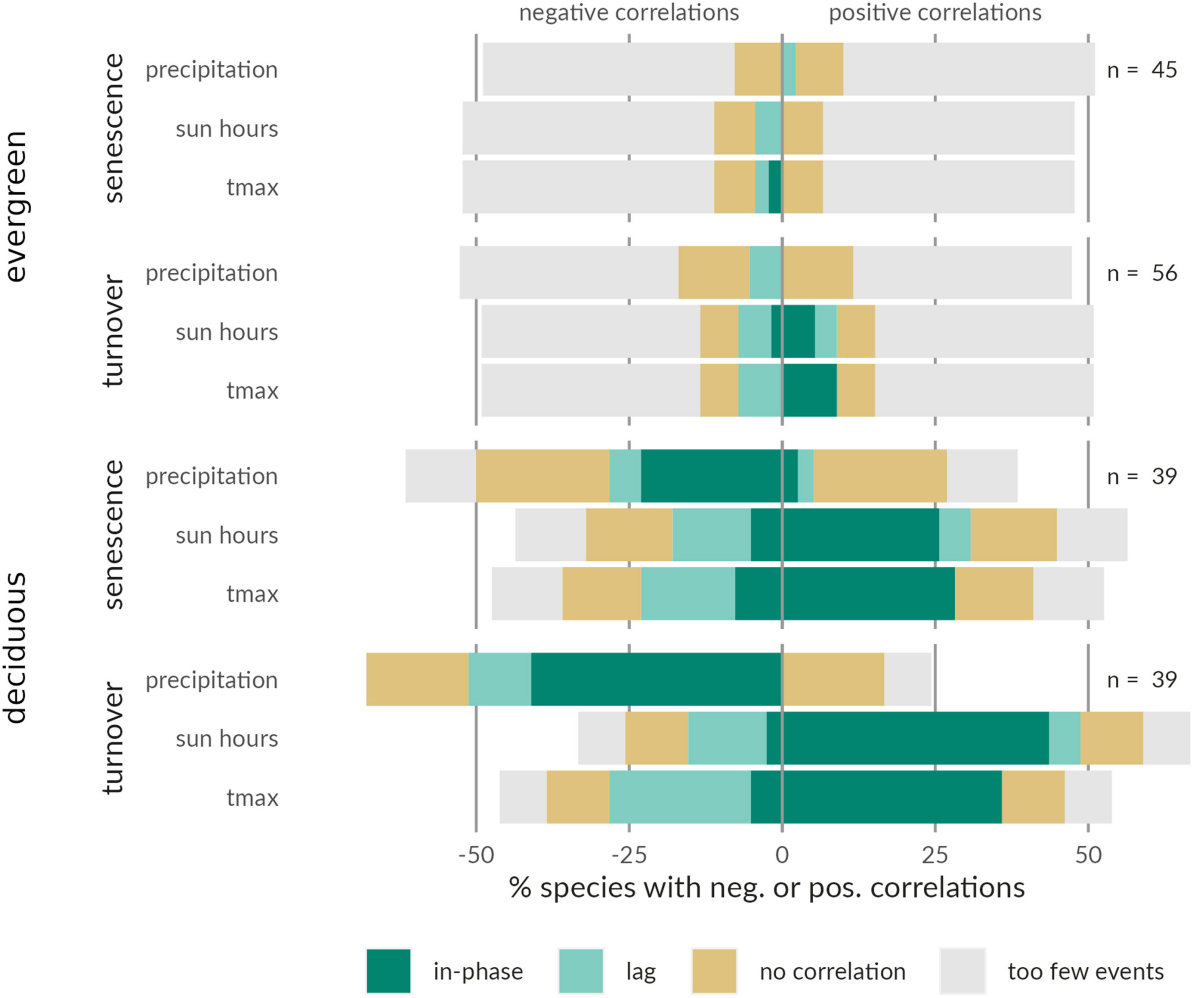
Overview of species‐specific correlations between phenological observations and climate variables precipitation, sun hours, and maximum temperature (tmax). For all evergreen and deciduous species with any phenophases observed, the percentage of species with negative (left) or positive (right) in‐phase relationships or phenophases lagging in time in relation to climatological cues are shown (*p* < .05). The percentage of species for which no correlations are found or for which too few observations were observed to investigate their relationship with the climate variables are divided over both sides. Each full bar shows 100% of the investigated species with the total number of species indicated as *n*.

Time‐series cross‐correlations between species‐level phenological observations and in situ measured monthly precipitation, sun hours, and maximum temperature revealed several significant relationships (*p* < .05; Figure 3; Table 1; Tables S2 and S3). The leaf phenophases for 23%–44% of the deciduous species were related to periods of limited rainfall, large amounts of sun hours, and warm temperatures, which corresponds to the long dry season (Dec–Feb; Figure 2, right panels). Similar relationships with the same climate conditions were found for canopy turnover (36%–44%) and senescence (23%–28%). A considerable number of other deciduous species showed phenophase periods following months (i.e., lagged responses) with lower amounts of sun hours and lower temperatures (senescence: 13% and 15%; turnover: 13% and 23%), conditions typical for the beginning of the long wet season (~Aug). In contrast, fewer significant cross‐correlations with climate (*p* < .05) were found for evergreen species (Figure 3). The timing of canopy turnover for certain evergreen species was occasionally in‐phase with months experiencing a large number of sun hours (5%) and high temperatures (9%). While another 5%–7% of the evergreen species showed turnover events lagging periods of low rainfall, fewer hours of sunlight and/or lower temperatures by 1–2 months. Significant correlations (*p* < .05) for senescence were rare.

**FIGURE 4:**
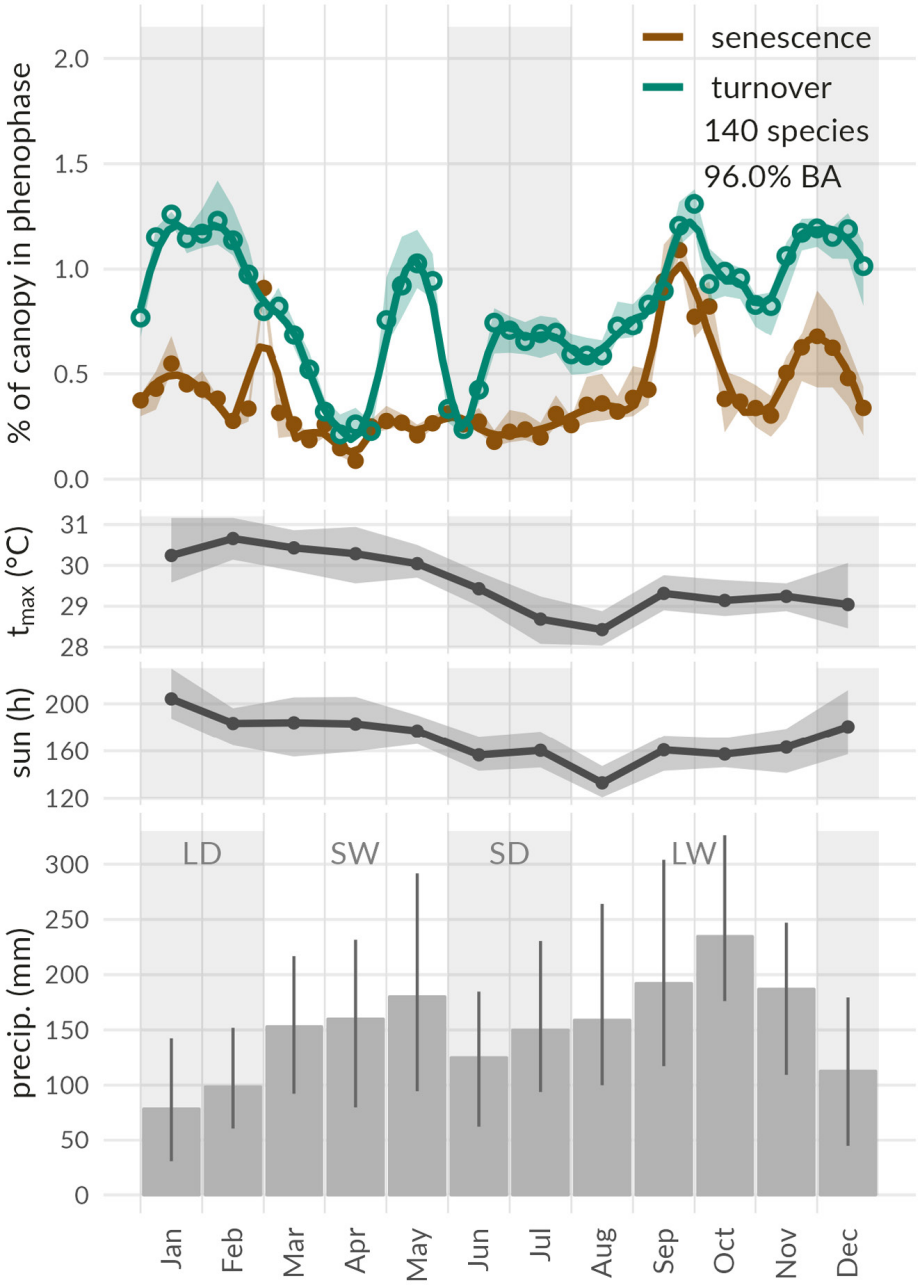
Average annual leaf phenological and meteorological patterns at the Yangambi forest reserve. Aggregated historical phenological patterns (1937–1956) of leaf senescence (brown, full points) and turnover (green, open points) are shown in the upper panel. All signals are combined annual species‐specific patterns weighted by their contribution to stand‐level basal area as a proxy for their contribution to the forest canopy. A total of 96.0% of the basal area is represented. Variability between the three inventories is indicated with shaded areas. The three lower panels show annual averages of in situ measured monthly maximum temperature (°C), sun hours, and precipitation (mm) during the phenological observational study (1951–1956 and 1937–1956, respectively). Gray‐shaded areas represent the average timing of the long and short dry seasons (LD and SD; monthly precipitation <150 mm), separated by the long and short wet seasons (LW and SW).

**FIGURE 5:**
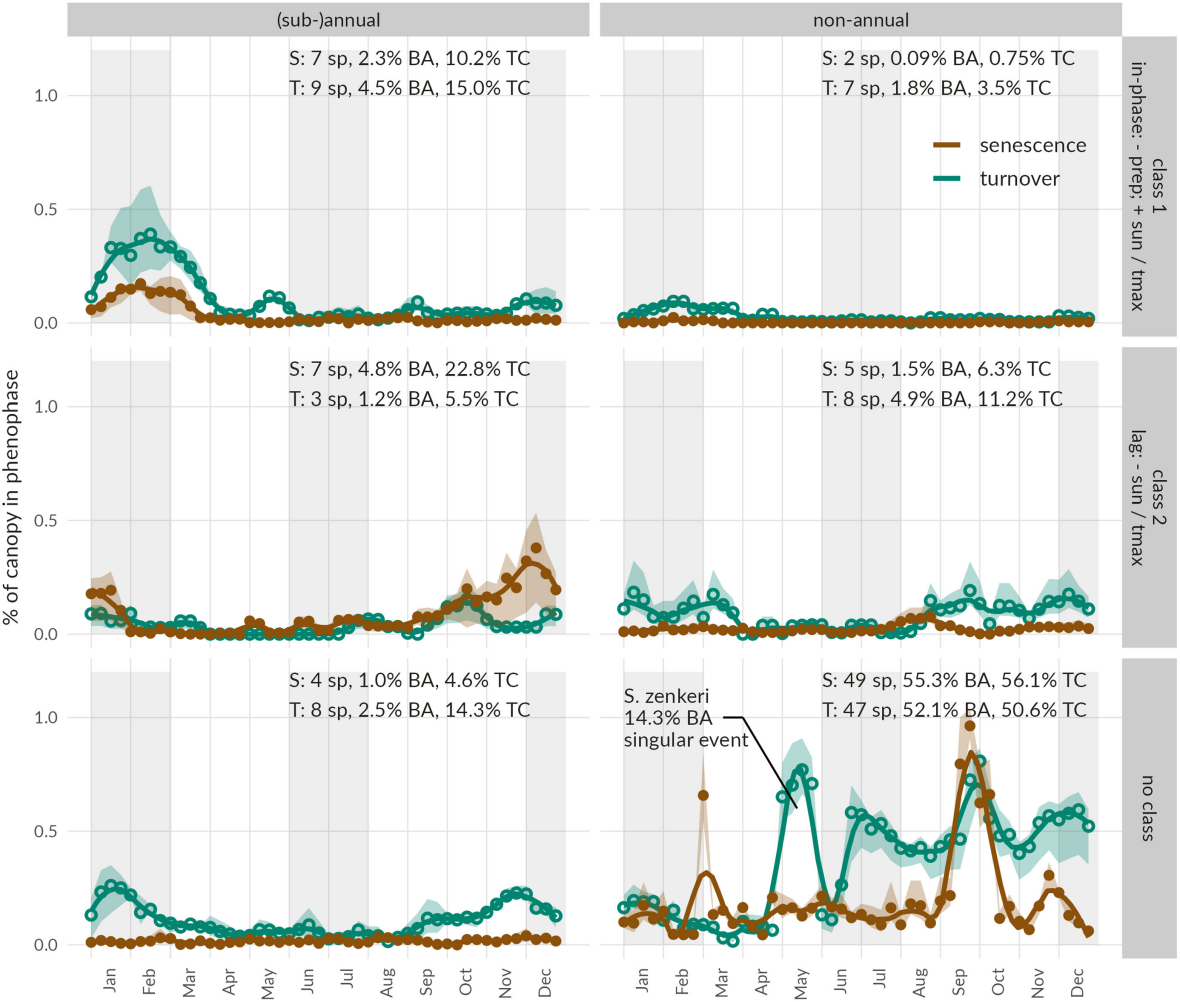
Average annual leaf phenological patterns of species groups characterized by the climate‐correlation and seasonality analysis. The columns represent groups of species with a (sub‐)annual or nonannual cyclicity and the rows show species groups with (1) a negative in‐phase correlation to precipitation and a positive in‐phase correlation to sun hours and maximum temperature (class 1), (2) a negative lagged correlation to sun hours and maximum temperature (class 2), and (3) the remainder of the species (no class). Group‐specific phenological patterns of senescence (brown, full points) and turnover (green, open points) are shown in the upper panel. All signals are combined annual species‐specific patterns weighted by their contribution to stand‐level basal area as a proxy for their contribution to the forest canopy (shaded areas show variability between the three inventories). In each panel, for both senescence (S) and turnover (T), respectively, the number of species (sp), cumulative percentage of basal area (BA), and the total contribution (TC) to the stand‐level signal (shown in Figure 4) is indicated. A peak specifically dominated by a single species with large basal area is indicated (*Scorodophloeus zenkeri*). Gray‐shaded areas represent the average timing of the long and short dry seasons (LD and SD; monthly precipitation <150 mm), separated by the long and short wet seasons (LW and SW).

Aggregating our historical species‐specific data together with basal area (BA) data we could scale to the canopy level. Aggregation demonstrated that only a small fraction of the canopy (~1%–1.5%) is in a state of senescence or turnover throughout the year (Figure 4). The stand‐level signal appears erratic and challenging to interpret. However, combining our cross‐correlation and Fourier‐based seasonality analysis we are able to distinguish six characteristic groups contributing to this stand‐level signal, irrespective of the classification as evergreen or deciduous (Figure 5). A first axis of classification (k‐means) through cross‐correlations identifies three main classes: class 1, species with phenophases related to periods of limited rainfall, large amounts of sun hours and warm temperatures, corresponding to the long dry season (Dec–Feb); and class 2, species with phenophases occurring after months of lower amounts of sun hours and lower temperatures, when these variables start rising again; corresponding to the middle of the long wet season (~Sep–Oct); and class 3: remaining species with no strong pattern, referred to as “no class.”

A second axis of classification through a Fourier‐based measure of seasonality further distinguishes the species into (sub‐)annual and nonannual classes, indicating how consistent they are with their contribution to the canopy‐level signal. Only a few species exhibited a (sub‐)annual cycle, with limited year‐on‐year variability in phenology. Yet these species with (sub‐)annual seasonality contribute disproportionately to our average stand‐level signal. In particular, while representing only 8.2% of the stand‐level basal area they contribute from 34.8% to 37.6% of the stand‐level phenological signal (Figure 5).

Species without annual seasonality that do show significant correlations to climate represent a lower but still considerable percentage of the average annual signal (senescence: 1.6% BA–7.1% pattern; turnover: 6.7% BA–14.7% pattern), although with higher interannual variability. Finally, species not showing any relation to climate (52.1%–55.3% BA) account for 56.1% of senescence and 50.6% turnover. This remainder of the stand‐level signal (Figure 5, no class) is less clear, with some abundant and large‐sized species (e.g., *S. zenkeri*) driving spikes in the signal. As these abundant or large‐sized species show no detectable pattern in their phenological signals, with many quiescent years without phenological activity, the amplitude, and timing of this compiled signal is less certain. This group thereby introduces considerable noise into the overall canopy signal.

Although clear grouped signals can be found, linked to seasonality and climate variables, our aggregation combining all data showed no significant “integrated canopy” correlations to any climate variables (i.e., *p* > .05) as it is composed of different groups of tree species that are responding very differently to particular seasonal climate cues.

## 4 DISCUSSION

The detailed long‐term historical phenological observations covering 140 tropical tree species in the central Congo Basin provide a rare opportunity to disentangle the phenological patterns that contribute to the overall turnover of a tropical forest canopy. Our analysis shows highly divergent behavior among species and considerable intraspecies and interannual variability (Figure 2). While Congo basin forest canopy dynamics derived from remotely sensed data products are strongly related to the seasonality in precipitation generated by the movement of the Intertropical Convergence Zone (Gond et al., 2013), our aggregated phenology signal does not match the strong bi‐modal component of EVI or rainfall when analyzed as a whole (Figure 4). However, we demonstrate that through particular groups of species, important components of the aggregated signal can be tied to climate variables, and EVI.

A large fraction of our aggregated signal exhibits synchronous annual phenology in senescence and turnover with (lagged) responses to climate drivers of either dry season (Figures 4 and 5). Phenological responses of specific tree species are hereby decoupled in time with two groups showing separate synchronized behavior. Phenology of the long dry season is associated with reduced precipitation, high light availability, and high temperatures, while lagged responses to the short dry season are driven by decreased light availability and temperatures. Despite the small number of species with annual phenological seasonality and their modest combined basal area (8%) they represent up to 38% of our aggregated canopy‐level phenology signal. These species have a disproportionate influence on canopy phenology, due to their synchronous nature. Additionally, particular species within diverse tropical forests can dominate the basal area due to the large size they attain or their high abundance within the community. Large or abundant trees not only have a disproportionate influence on carbon storage and productivity (Slik et al., 2013), but they can also influence the strength and level of uncertainty of the canopy‐wide phenology signal. This seems to be the case in Yangambi, where *S. zenkeri*, a dominant evergreen species shows few but dominant phenophase events that impact the large‐scale phenology signal (Figure 5). In this case, strong links with drivers are missing due to the sporadic nature of this species' phenophase events, while highly contributing to the variability and uncertainty in the canopy‐level phenological signal through its dominant presence.

Correlations between various drivers make it hard to discern endogenous (i.e., internal) and additional underlying exogenous (environmental) drivers of the remaining interannual variability. The strong annual phenological activity corroborates previous research suggesting endogenous control on rainforest leaf phenology (Reich, 1995), despite associations to climate drivers. Species not showing annual cyclicity are more directly related to climate across both dry seasons, with species‐specific phenological dynamics contrasting between both. Previous studies generally agree that when water availability is not limited, light availability is the main driving factor for tropical forest phenology (Adole et al., 2019; Bradley et al., 2011; Guan et al., 2015; Philippon et al., 2016). However, drought‐related stress does appear to be an important cue for leaf senescence for a portion of the species investigated (~6.5% of species assessed, representing 2.7% BA). African tropical forests are reported to be more drought‐stressed than Amazonian or Asian forests (Malhi & Wright, 2004) and seem to be primarily controlled by rainfall (Gond et al., 2013; Guan et al., 2015). Yet, a larger proportion of the species do show an in‐phase correlation to light and temperature (~21% of species assessed, representing 11.3% BA). Temperature is generally considered to not limit growth in tropical forests (Nemani et al., 2003), and seasonality in sun hours is probably more important (Adole et al., 2019; Philippon et al., 2016, 2019). Indeed, changes in light availability and corresponding changes in leaf demography are key to optimizing forest productivity by maximizing light availability for photosynthesis (Caldararu et al., 2014; Restrepo‐Coupe et al., 2017). However, compared to the Amazon (Wu et al., 2016) the relationship between light availability and precipitation is less straightforward in the Congo Basin, as sun hours differ across both dry seasons (Figure 4; Philippon et al., 2016). This split in light availability across the two dry seasons widens the potential phenological strategies to optimize productivity through more diverse climate niches. Additionally, remote sensing analysis has shown that west‐central African forests remain evergreen through extensive low‐level cloudiness during the dry season, limiting evapotranspiration (Philippon et al., 2019). However, to corroborate any strong links between exogeneous leaf phenology, productivity and biodiversity, long‐term in situ gas exchange measurements (Dötterl et al., 2020), and observations of tree growth (de Mil et al., 2019) and phenology (Abernethy et al., 2018) would be needed.

The high phenological diversity found at Yangambi is not unexpected as tropical phenological events can often be relatively asynchronous within and among different species co‐existing in the same communities and experiencing a broadly similar climate (Reich, 1995; Reich et al., 2004; van Schaik et al., 1993). While we show climatic variables cause a significant convergence in phenophases across some species, for a large number of species, both deciduous and evergreen, phenophase patterns remain unexplained by climate alone (Figure 3). Multiple other drivers and processes, both endogenous and exogenous, are potentially at play in controlling the timing of leaf developmental stages. These can include niche complementarity as an important means to optimize light capture across both space and time (Sapijanskas et al., 2014), variations in the micro‐habitats of individual trees (e.g., different degrees of competition with neighboring trees), ontological changes caused by changing light environment for (non‐)emergent species (Reich et al., 2004) and leaf flushing during periods of minimal insect activity and fungal pathogen pressure (Wright & Calderón, 1995). We cannot speculate on the importance of these cues for the investigated species yet, but the high variability found among species suggests they should not be overlooked.

Although we can explain some of the variability in the canopy‐level phenology, uncertainty remains. Approximately 56% of the variability in the aggregated signal shows little coherence with any climate variable—contrary to contemporary literature on leaf phenology expectations (evergreen, [brevi‐]deciduous, see methodology for references). More so, for 22% of the investigated species no leaf phenophases were observed and an additional 21% of species showed too few observations (<5 observed leaf phenophases) across individuals and years to analyze their seasonality. It is assumed that these species have a continuous, gradual leaf turnover that is not easy to follow using ground‐based canopy observation techniques (Reich et al., 2004). This continuous canopy activity (Albert et al., 2019) is not accounted for in our stand‐level signal.

**FIGURE 6:**
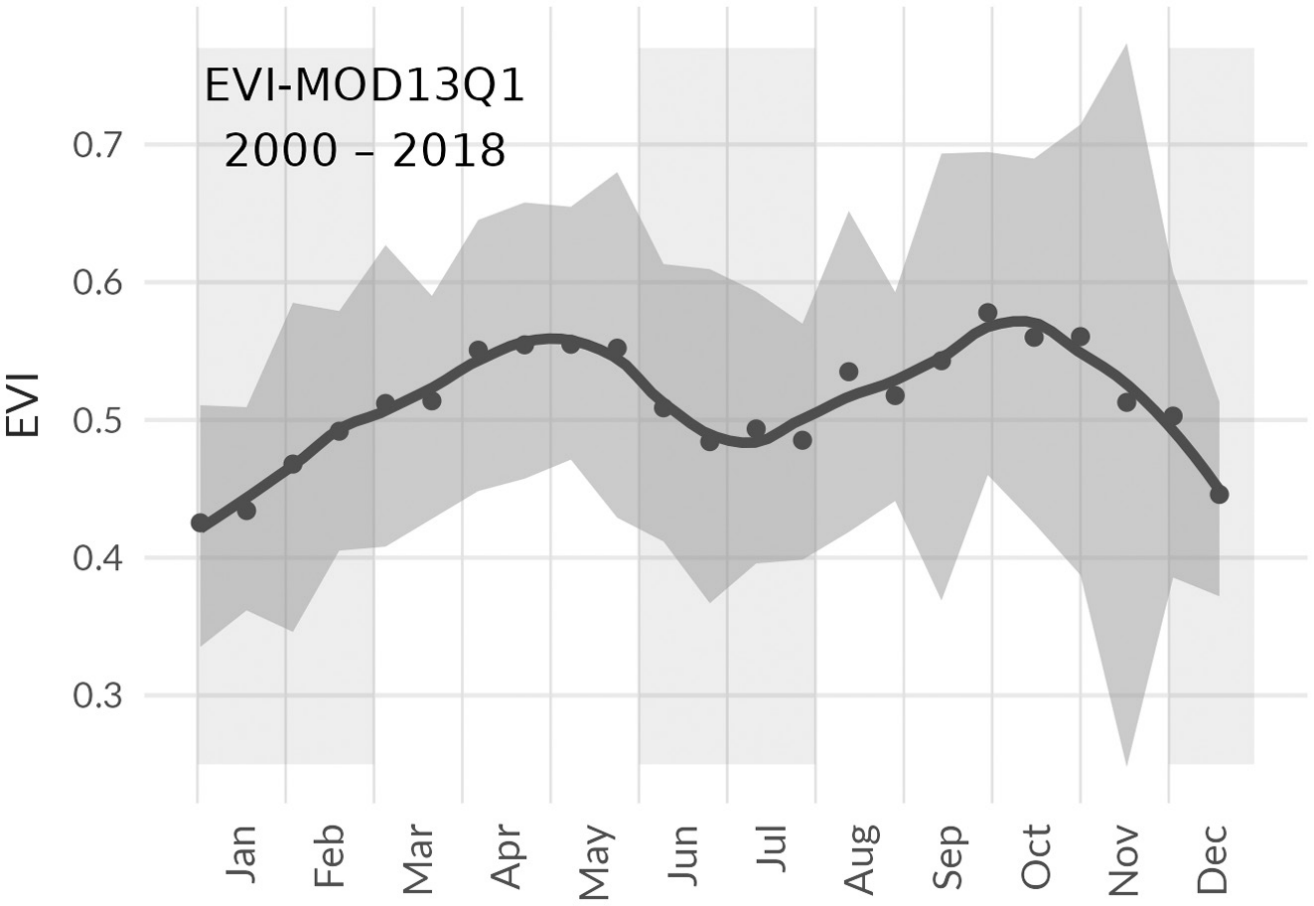
The annual mean profile of EVI‐MOD13Q1 (2000–2018; Didan, 2015) for the study area in Yangambi, with the shaded area again indicating plot‐level variability.

Contemporary analysis of Congo basin canopy dynamics and phenology are generally based on remotely sensed data products such as MODIS EVI (Figure 6). While components of our aggregated canopy‐level phenology signal based on historical observations show similarities to remotely sensed patterns, such as the correspondence of the annual phenological activity of a group of species to the largest shift in EVI throughout the year, the patterns are not directly comparable. Methodological considerations are required when comparing the scaled canopy phenology signals with those presented from EVI. The presence and absence of leaves in a canopy are not the sole drivers that make up the EVI signal, as it is composed of both structural and biochemical information. Thus, it is not surprising that the ground‐based phenophase patterns of senescence and turnover at the canopy scale do not fully capture the EVI signal, even though some species groups did follow the general seasonal trends in EVI. We can only speculate that the time lag in the main period of leaf flushing at the end of the dry season and the major peak in EVI that followed shortly afterward in the wet season could be linked to the maturation of the recently flushed leaves and the upregulation of photosynthetic pigments as the rainy season progresses (Doughty & Goulden, 2008; Restrepo‐Coupe et al., 2013; Wu et al., 2016, 2017). Another reason for discrepancies between both signals is the obvious time lag of 60+ years between both data sets, where one is dated largely before the large‐scale impacts of climate change (Lewis & Maslin, 2015). Both global temperatures and CO_2_ concentrations have risen by 0.82° (2019–1958, GISTEMP 2020; Lenssen et al., 2019) and ~95 ppm (NOAA‐GML, n.d.), respectively, influencing phenology (Parmesan & Yohe, 2003; Schwartz et al., 2006; Wang et al., 2016) and (leaf) physiology alike (Bauters et al., 2020; Beerling et al., 1998). As such, we can not rule out extant shifts in EVI phenology due to climate change. Yet, given the lack of contemporary tropical leaf phenology data compounded by the lack of historic satellite data, it is necessary to assume that these aggregated signals can be compared.

Moreover, discrepancies between the historical and contemporary phenological signals are expected to increase. For example, a recent study shows that tropical forests are close to exceeding their temperature optima (Huang et al., 2019) and increasing their dry season length (Jiang et al., 2019), which might shift species into an unfamiliar and unfavorable domain. As such the influence of drought‐driven seasonality on tropical vegetation phenology (enforced by physiological limits) could become more important (Jiang et al., 2019). Uncertainty in climate re‐analysis driver data, for example, temperature and precipitation, combined with the reliance on large‐scale contemporary remotely sensed data, limits modeling exercises of species‐specific and ecosystem scale responses (Chen et al., 2017; Wright & Calderón, 2018). Future modeling efforts with high‐resolution in situ climate (Jacobsen et al., 2018) could provide the opportunity to identify which species are likely to respond to these changes in climate, capturing influences on productivity and biodiversity. Here, our preclimate change phenological responses can serve as a reference for such studies, providing a long‐term view of ecosystem changes.

The unique insights obtained from our study demonstrate that the large diversity in species‐specific phenological patterns dampens within‐year variability at the ecosystem scale, even though some species exhibited pronounced synchronous phenophases at different times. Even though central African forests experience relatively low rainfall compared with other tropical forests, the majority of the species still exhibit evergreen phenology. Furthermore, our canopy‐level aggregation suggests that along the year, a maximum of 1.5% of the canopy is in phenophase. The overall phenological variability, however, is largely dominated by a relatively low amount of deciduous species that are triggered by climate (precipitation, temperature, and sun hours). Overall, the emergent canopy‐level pattern derived from in situ observations did not correspond directly to the bi‐modal EVI signal, contrary to the pattern generally observed in temperate ecosystems between leaf phenology and EVI (Hufkens et al., 2012). In contrast to temperate ecosystems, tropical ecosystems display a decoupling between the phenology signals of structural components in a canopy and those of functional components driven by an increase in metabolites particularly pigments (Doughty & Goulden, 2008; Restrepo‐Coupe et al., 2013; Wu et al., 2016, 2017). Integrated measurements of both structural and functional components across a wide variety of species will be necessary to fully capture phenological processes, their relation to climate niches, and influence on adaptation and biodiversity.

## 5 CONCLUDING REMARKS

This long‐term phenological record, before large‐scale impacts of climate change, forms an important baseline reference for central African tropical forests. Our analysis emphasizes the high variability found in leaf phenological patterns and the various drivers at play, be it endogenous or exogenous. As such, historical baseline variability and variability in drivers of tropical tree leaf phenology need to be considered when informing global dynamic vegetation models, especially before further predictions on the impact of climate change are made. A first step to increase model accuracy would be the partitioning of species according to broad phenological characteristics tied to primary environmental drivers. Yet, a significant amount of phenological behavior remains unexplained due to high interannual and intra‐ and interspecies variability. This variability precludes quantifying leaf phenological patterns using medium‐resolution remote sensing products without detailed knowledge of underlying stand‐level phenological patterns driven by species composition, even without considering the impact of environmental cues. Contemporary ground‐based long‐term monitoring with high coverage of species in tropical forests is needed to understand both historical and current phenological behavior and how these multi‐species ecosystems scale to the landscape level in terms of phenology.

## CONFLICT OF INTEREST STATEMENT

The authors declare no conflicts of interest.

## DATA AVAILABILITY STATEMENT

The manuscript's database supporting our findings is made available in a Zenodo Digital Repository (Kearsley et al., 2023). The code used during the analysis of the “Jungle Rhythms” project is available as an R (R Core Team, 2020) project at https://github.com/bluegreen‐labs/junglerhythms/ (Hufkens & Kearsley, 2023). The analysis relied on the “circular” (Agostinelli & Lund, 2017), “stats” (R Core Team, 2020) and “tseries” (Trapletti & Hornik, 2019) packages, while post‐processing and plotting was facilitated by the “tidyverse” ecosystem (Wickham et al., 2019), “dplyr” (Wickham et al., 2020), and “ggthemes” (Arnold, 2019) packages. We are grateful for the contributions by the developers of these packages to the scientific community.

## Supporting information


Data S1:
