# Supplementary material for: Historical tree phenology data reveal the seasonal rhythms of the Congo Basin rainforest
Source: Plant Environ Interact. 2024 Mar 11;5(2):e10136. doi: 10.1002/pei3.10136 (PMC10926959; doi:10.1002/pei3.10136)

Supplementary Information - Historical tree phenology data reveal the seasonal rhythms of the Congo Basin rainforest

Table S1: Overview of all 140 investigated species. Full species names including families are provided. Phenological classifications and shade tolerance are based on a literature search of published floras (Arbonnier 2004, Hawthorne & Jongkind 2006, Lemmens et al. 2012, Meunier et al. 2015, Meerts & Hasson 2016). Species classified as 'evergreen or deciduous' in literature are reclassified based on the historical phenological observations, indicated with a single asterisk (*). Species with phenology indicated by a double asterisk (**) were not reported in literature and classification is based on the historical phenological observations. Species representation in the forest composition is represented as the percentage basal area (BA) of the historical forest inventory in Yangambi (Ygb, Pierlot 1966). Species-specific data availability in the phenological archives is provided as the number of individuals (ind) observed and the total amount of observation (obs) years available.

		characteristics literature		Pierlot 1966	archives	
**species**	**family**	**phenology**	**ecology**	**% BA**	**nr ind**	**nr obs years**
Afrostyrax lepidophyllus Mildbr.	Huaceae	evergreen	shade	0.21	5	27
Albizia adianthifolia (Schumach.) W. Wight	Leguminosae	deciduous	sun	0.26	7	57
Albizia ferruginea (Guill. & Perr.) Benth.	Leguminosae	deciduous		0.18	10	70
Allanblackia floribunda Oliv.	Clusiaceae	evergreen	shade	0.15	4	28
Allophylus africanus P. Beauv.	Sapindaceae	deciduous**		0.06	7	38
Alstonia boonei De Wild.	Apocynaceae	deciduous	sun	0.16	1	1
Amphimas pterocarpoides Harms	Leguminosae	deciduous		0.13	9	70
Angylocalyx pynaertii De Wild.	Leguminosae	evergreen	shade	0.9	7	41
Anonidium mannii (Oliv.) Engl. & Diels	Annonaceae	evergreen	shade	5.16	3	31
Anthonotha macrophylla P. Beauv.	Leguminosae	deciduous	sun-shade	0.2	7	63
Antiaris toxicaria (Engl.) C.C. Berg	Moraceae	deciduous		0.12	2	7
Antrocaryon nannanii De Wild.	Anacardiaceae	deciduous	sun	0.2	5	52
Baikiaea insignis Benth.	Leguminosae	evergreen		0.23	8	34
Baphia laurifolia Baill.	Leguminosae	deciduous		0.11	3	13
Baphia pubescens Hook. f.	Leguminosae	evergreen		0.39	4	15
Barteria fistulosa Mast.	Passifloraceae	evergreen	sun	0.07	1	3
Beilschmiedia louisii Robyns & R. Wilczek	Lauraceae	evergreen		1.45	4	42
Berlinia grandiflora (Vahl) Hutch. Dalziel	Leguminosae	deciduous		0.04	3	30
Blighia welwitschii (Hiern) Radlk	Sapindaceae	evergreen	shade	2.06	8	58
Brachystegia laurentii (De Wild.) Hoyle	Leguminosae	deciduous*	shade	0.82	3	5
Caloncoba welwitschii (Oliv.) Gilg	Achariaceae	deciduous		0.07	1	8
Canarium schweinfurthii Engl.	Burseraceae	deciduous		0.08	5	40
Carapa procera DC.	Meliaceae	evergreen	shade	0.16	9	56
Celtis mildbraedii Engl.	Cannabaceae	evergreen*	shade	1.35	9	57
Celtis tessmannii Rendle	Cannabaceae	deciduous	sun-shade	0.73	6	40
Chlamydocola chlamydantha (K. Schum.) M. Bodard	Malvaceae	evergreen**		0.1	3	11
Chrysophyllum africanum A. DC.	Sapotaceae	evergreen	shade	0.59	4	33
Chrysophyllum lacourtianum De Wild.	Sapotaceae	evergreen	shade	1.58	4	48
Chrysophyllum pruniforme Engl.	Sapotaceae	evergreen	shade	0.06	2	17
Cleistanthus polystachyus Hook. f. ex Planch.	Phyllanthaceae	-		0.62	1	2
Coelocaryon preussii Warb.	Myristicaceae	evergreen	shade	0.13	5	48
Cola griseiflora De Wild.	Malvaceae	evergreen	shade	4.77	8	62
Cola lateritia K. Schum.	Malvaceae	deciduous	shade	0.5	7	57
Colletoecema dewevrei (De Wild.) E.M.A. Petit	Rubiaceae	-		0.05	1	2
Combretum lokele Liben	Combretaceae	deciduous*	shade	2.12	1	7
Copaifera mildbraedii Harms	Leguminosae	deciduous**	sun-shade	0.28	4	26
Croton mubango Müll. Arg.	Euphorbiaceae	evergreen*		0.04	1	13
Cynometra alexandri C.H. Wright	Leguminosae	evergreen	shade	0.79	2	2
Cynometra hankei Harms	Leguminosae	evergreen	sun-shade	2.04	5	30
Dacryodes edulis (G. Don) H.J. Lam	Burseraceae	evergreen	sun-shade	0.15	4	27
Dacryodes osika (Guillaumin) H.J. Lam.	Burseraceae	evergreen	sun-shade	0.05	4	43
Dactyladenia dewevrei (De Wild. & T. Durand) Prance & F. White	Chrysobalanaceae	evergreen**		0.15	3	12
Desplatsia dewevrei (De Wild. & T. Durand) Burret	Malvaceae	evergreen	sun	0.23	6	36
Dialium corbisieri Staner	Leguminosae	evergreen**		0.96	1	7
Dialium excelsum Steyaert	Leguminosae	deciduous	shade	0.27	11	76
Dialium pachyphyllum Harms	Leguminosae	evergreen	shade	1.1	7	47
Dialium pentandrum Steyaert	Leguminosae	-		0.13	3	23
Dichostemma glaucescens Pierre	Euphorbiaceae	evergreen**		0.26	4	18
Diogoa zenkeri (Engl.) Exell & Mendona	Olacaceae	evergreen**		0.11	3	9
Diospyros crassiflora Hiern	Ebenaceae	evergreen	shade	0.91	4	38

Table S1 continued

species	family	characteristics literature		Pierlot 1966	archives	
		phenology	ecology	% BA	nr ind	nr obs years
Diospyros hoyleana F. White	Ebenaceae	evergreen		0.04	7	50
Diospyros iturensis (Gürke) Letouzey & F. White	Ebenaceae	evergreen	shade	0.65	9	84
Discoglypremna caloneura (Pax) Prain	Euphorbiaceae	deciduous	sun	0.09	2	18
Drypetes gossweileri S. Moore	Putranjivaceae	evergreen	shade	1.45	5	29
Drypetes leonensis Pax	Putranjivaceae	-		0.09	1	3
Drypetes spinosodentata (Pax) Hutch.	Putranjivaceae	evergreen**		0.85	4	28
Entandrophragma angolense (Welw.) C. DC.	Meliaceae	deciduous	sun-shade	0.08	5	38
Entandrophragma candollei Harms	Meliaceae	deciduous	sun-shade	0.52	8	50
Entandrophragma cylindricum (Sprague) Sprague	Meliaceae	deciduous	sun-shade	0.6	9	84
Eriocoelum microspermum Radlk. ex Engl.	Sapindaceae	evergreen	shade	0.17	5	40
Erythrophleum suaveolens (Guill. & Perr.) Brenan	Leguminosae	deciduous	sun	1.26	5	40
Fernandoa adolfi-friderici Gilg & Mildbr.	Bignoniaceae	deciduous**		0.36	7	61
Ficus ardisioides Warb.	Moraceae	evergreen		0.17	1	5
Funtumia africana (Benth.) Stapf	Apocynaceae	evergreen	shade	0.04	1	1
Garcinia epunctata Stapf	Clusiaceae	evergreen	shade	0.26	2	19
Garcinia punctata Oliv.	Clusiaceae	evergreen	shade	1.09	5	37
Garcinia smeathmanii (Planch. & Triana) Oliv.	Clusiaceae	evergreen	shade	0.44	7	36
Gilbertiodendron dewevrei (De Wild.) J. Léonard	Leguminosae	evergreen	shade	0.81	8	45
Gilletiodendron mildbraedii (Harms) Vermoesen	Leguminosae	evergreen**	sun-shade	0.14	5	38
Greenwayodendron suaveolens (Engl. & Diels) Verdc.	Annonaceae	deciduous	shade	5.73	2	18
Guarea cedrata (A. Chev.) Pellegr.	Meliaceae	evergreen	shade	1.06	1	8
Hymenocardia ulmoides Oliv.	Phyllanthaceae	evergreen**		0.07	7	34
Irvingia gabonensis (Aubry-Lecomte ex O'Rorke) Baill.	Irvingiaceae	-	shade	0.56	1	13
Irvingia grandifolia (Engl.) Engl.	Irvingiaceae	deciduous	sun-shade	0.49	7	70
Isolona hexaloba (Pierre) Engl. & Diels	Annonaceae	evergreen**		0.32	7	84
Klainedoxa gabonensis Pierre	Irvingiaceae	deciduous	sun	0.44	8	71
Lasiodiscus fasciculiflorus Engl.	Rhamnaceae	deciduous		0.31	1	5
Leplaea laurentii (De Wild.) E.J.M. Koenen & J.J.de Wilde	Meliaceae	evergreen**		1.93	5	53
Macaranga barteri Müll. Arg.	Euphorbiaceae	evergreen	sun	0.05	3	21
Macaranga monandra Müll. Arg.	Euphorbiaceae	-		0.34	3	9
Mammea africana Sabine	Calophyllaceae	evergreen	shade	0.12	11	64
Maranthes glabra (Oliv.) Prance	Chrysobalanaceae	evergreen	shade	0.08	2	10
Massularia acuminata (G. Don) Bullock ex Hoyle	Rubiaceae	evergreen	shade	0.15	2	5
Microdesmis puberula Hook. f. ex Planch.	Pandaceae	evergreen		0.58	4	33
Milicia excelsa (Welw.) C.C.Berg	Moraceae	deciduous	sun	0.05	11	97
Monodora myristica (Gaertn.) Dunal	Annonaceae	deciduous	shade	0.16	3	21
Myrianthus arboreus P. Beauv.	Urticaceae	evergreen	sun	0.07	5	18
Napoleonaea vogelii Hook. & Planch.	Lecythidaceae	evergreen**		0.07	4	36
Nauclea diderrichii (De Wild. & T. Durand) Merr.	Rubiaceae	deciduous*		0.14	7	49
Newtonia devredii G.C.C. Gilbert & Boutique	Leguminosae	-		0.04	1	2
Omphalocarpum ghesquierei De Wild.	Sapotaceae	evergreen**		0.23	1	13
Ongokea gore (Hua) Pierre	Olacaceae	evergreen	shade	0.85	6	66
Pancovia harmsiana Gilg	Sapindaceae	evergreen	shade	0.1	2	19
Pancovia laurentii (De Wild.) Gilg ex De Wild.	Sapindaceae	evergreen	shade	1.02	7	62
Panda oleosa Pierre	Pandaceae	evergreen	shade	2.32	5	61
Paramacrolobium coeruleum (Taub.) J. Léonard	Leguminosae	evergreen	sun	0.26	2	20
Parinari excelsa Sabine	Chrysobalanaceae	evergreen	sun-shade	0.19	1	5
Parkia bicolor A. chev.	Leguminosae	deciduous		0.1	3	15
Pausinystalia macroceras (K. Schum.) Pierre ex Beille	Rubiaceae	evergreen	shade	0.09	4	31
Pentaclethra macrophylla Benth.	Leguminosae	deciduous*	sun-shade	0.81	5	46
Pericopsis elata (Harms) Meeuwen	Leguminosae	deciduous**	sun	0.76	5	50
Petersianthus macrocarpus (P. Beauv.) Liben	Lecythidaceae	deciduous	sun-shade	1.17	3	26
Phyllocosmus africanus (Hook. f.) Klotzsch	Ixonanthaceae	evergreen	shade	0.28	9	45
Platysepalum chevalieri Harms	Leguminosae	evergreen**		0.09	3	19
Pleiocarpa pycnantha (K. Schum.) Stapf	Apocynaceae	evergreen	shade	0.51	5	31
Prioria balsamifera (Vermoesen) Breteler	Leguminosae	evergreen	shade	1.1	8	38
Prioria oxyphylla (Harms) Breteler	Leguminosae	evergreen	sun-shade	2.36	10	94
Pteleopsis hylodendron Mildbr.	Combretaceae	deciduous		0.3	8	36
Pterocarpus soyauxii Taub.	Leguminosae	deciduous	sun-shade	1.5	6	45
Pterygota bequaertii De Wild.	Malvaceae	deciduous		0.37	8	73
Pycnanthus angolensis (Welw.) Warb.	Myristicaceae	evergreen	sun-shade	0.11	5	43
Quassia silvestris Cheek & Jongkind	Simaroubaceae	evergreen*	sun	0.32	6	52
Radlkofera calodendron Gilg	Sapindaceae	evergreen**	sun	0.04	5	32
Ricinodendron heudelotii (Baill.) Pierre ex Heckel	Euphorbiaceae	deciduous	sun	0.04	7	60
Rinorea welwitschii (Oliv.) Kuntze	Violaceae	deciduous		0.14	2	13
Scorodophloeus zenkeri Harms	Leguminosae	evergreen	shade	14.76	4	27
Staudtia kamerunensis (Warb.) Fouilloy	Myristicaceae	evergreen	shade	0.94	4	38
Sterculia dawei Sprague	Malvaceae	deciduous	shade	0.14	5	61
Strombosia grandifolia Hook. f.	Olacaceae	evergreen	shade	0.74	8	85

Table S1 continued

		characteristics literature		Pierlot 1966	archives	
species	family	phenology	ecology	% BA	nr ind	nr obs years
Strombosia pustulata Oliv.	Olacaceae	evergreen	shade	1.64	5	48
Strombosiopsis tetrandra Engl.	Olacaceae	evergreen	shade	2.52	7	47
Symphonia globulifera L.f.	Clusiaceae	evergreen	shade	0.09	8	70
Synsepalum msolo (Engl.) T.D. Penn.	Sapotaceae	evergreen		0.1	5	31
Synsepalum subcordatum De Wild.	Sapotaceae	evergreen	shade	1.11	8	41
Syzygium congolense Vermoesen	Myrtaceae	-		0.12	2	5
Tabernaemontana crassa Benth.	Apocynaceae	evergreen**	sun-shade	0.18	1	10
Tessmannia anomala (Micheli) Harms	Leguminosae	evergreen	shade	0.04	3	30
Tetrorchidium didymostemon (Baill.) Pax & K. Hoffm.	Euphorbiaceae	evergreen	sun	0.05	5	33
Treculia africana Decne. ex Trcul	Moraceae	evergreen	sun-shade	0.05	5	44
Tricalysia pallens Hiern	Rubiaceae	evergreen	shade	0.06	1	10
Trichilia gilgiana Harms	Meliaceae	evergreen	shade	0.18	2	13
Trichilia prieuriana A. Juss.	Meliaceae	evergreen	shade	1.24	4	32
Trichilia rubescens Oliv.	Meliaceae	evergreen	shade	0.73	6	43
Tridesmostemon omphalocarpoides Engl.	Sapotaceae	evergreen**	shade	0.31	5	60
Trilepisium madagascariense DC.	Moraceae	evergreen		0.07	4	44
Turraeanthus africanus (Welw. ex C. DC.) Pellegr.	Meliaceae	evergreen	sun-shade	0.08	7	48
Vitex congolensis De Wild. & T. Durand	Lamiaceae	deciduous**		0.38	3	31
Xylopia aethiopica (Dunal) A. Rich.	Annonaceae	evergreen		0.05	8	50
Xylopia cupularis Mildbr.	Annonaceae	evergreen	sun	0.05	1	13
Zanthoxylum gilletii (De Wild.) P.G. Waterman	Rutaceae	deciduous		0.12	10	82

Table S2: Species-specific characteristics of the phenological cycles of leaf senescence. For each species, the number of individuals observed, the total observation years, the percentage of observation years with observed phenophases and the average duration (± SD) of a phenophase in weeks are indicated. Variability in the timing of phenophases across years is represented as inter-annual SD and within species (across individuals) as intra-species SD. Timing of fourier-based cyclicity is indicated. Significant correlations of time-series cross-correlation analysis between phenological observations and precipitation, amount of sun hours and maximum temperature are provided, with time of lag of the phenological observations in relation to each climatological variable indicated. Time t0 indicates significant in-phase cross-correlations ($p<0.05$); t-x indicates the time-lag in months of significant lagged cross-correlations ($p<0.05$).

species	nr ind	nr obs years	% years with phase	duration phase (w)	variability (SD)		cyclicity	cross-correlations		
					intra-sp.	inter-ann.		precip	sun hours	tmax
**evergreen**										
Afrostyrax lepidophyllus	5	27	3.7	2.2						
Allanblackia floribunda	4	28	0.0							
Angylocalyx pynaertii	7	41	7.3	1.4 ± 0.6	6.9					
Anonidium mannii	3	31	0.0							
Baikiaea insignis	8	34	0.0							
Baphia pubescens	4	15	13.3	1.4 ± 0.6	18.7					
Barteria fistulosa	1	3	0.0							
Beilschmiedia louisii	4	42	9.5	1.4 ± 0.5	6.3	4.4				
Blighia welwitschii	8	58	15.5	2.9 ± 2.2	16.9	12.8				
Carapa procera	9	56	5.4	9 ± 10.2	6.1	6.1				
Celtis mildbraedii	9	57	0.0							
Chlamydocola chlamydantha	3	11	0.0							
Chrysophyllum africanum	4	33	3.0	1.1						
Chrysophyllum lacourtianum	4	48	0.0							
Chrysophyllum pruniforme	2	17	0.0							
Coelocaryon preussii	5	48	4.2	2.7 ± 0.8						
Cola griseiflora	8	62	4.8	1.8 ± 0.6	11.3					
Croton mubango	1	13	0.0							
Cynometra alexandri	2	2	0.0							
Cynometra hankei	5	30	3.3	2.2						
Dacryodes edulis	4	27	3.7	1.1						
Dacryodes osika	4	43	11.6	2 ± 0.9	11.4					
Dactyladenia dewevrei	3	12	0.0							
Desplatsia dewevrei	6	36	0.0							
Dialium corbisieri	1	7	0.0							
Dialium pachyphyllum	7	47	0.0							
Dichostemma glaucescens	4	18	5.6	2.2						
Diogoa zenkeri	3	9	0.0							
Diospyros crassiflora	4	38	0.0							
Diospyros hoyleana	7	50	2.0	2.2						
Diospyros iturensis	9	84	1.2	6.5						
Drypetes gossweileri	5	29	3.4	1.1						
Drypetes spinosodentata	4	28	0.0							
Eriocoelum microspermum	5	40	0.0							
Ficus ardisioides	1	5	0.0							
Funtumia africana	1	1	0.0							
Garcinia epunctata	2	19	0.0							
Garcinia punctata	5	37	0.0							
Garcinia smeathmanii	7	36	0.0							
Gilbertiodendron dewevrei	8	45	2.2	2.2						
Gilletiodendron mildbraedii	5	38	2.6	3.2						
Guarea cedrata	1	8	0.0							
Hymenocardia ulmoides	7	34	8.8	2.9 ± 0.6	11.8					
Isolona hexaloba	7	84	0.0							
Leplaea laurentii	5	53	0.0							
Macaranga barteri	3	21	4.8	2.2						
Mammea africana	11	64	1.6	1.1						
Maranthes glabra	2	10	0.0							
Massularia acuminata	2	5	0.0							
Microdesmis puberula	4	33	0.0							
Myrianthus arboreus	5	18	0.0							
Napoleonaea vogelii	4	36	0.0							
Omphalocarpum ghesquierei	1	13	0.0							
Ongokea gore	6	66	3.0	3.8 ± 3.8						
Pancovia harmsiana	2	19	5.3	3.2						
Pancovia laurentii	7	62	3.2	2.2 ± 1.5						
Panda oleosa	5	61	1.6	2.2						

Table S2 continued

species	nr ind	nr obs years	% years with phase	duration phase (w)	variability (SD)		cyclicity	cross-correlations		
					intra-sp.	inter-ann.		precip	sun hours	tmax
**evergreen**										
Paramacrolobium coeruleum	2	20	15.0	2.2 ± 1.1	0.5					
Parinari excelsa	1	5	0.0							
Pausinystalia macroceras	4	31	12.9	2.2 ± 0.9	17.7	14.1				
Phyllocosmus africanus	9	45	15.6	2.5 ± 1.5	9.3			0.131 (t-1)	-0.15 (t-1)	-0.154 (t0)
Platysepalum chevalieri	3	19	10.5	4.9 ± 2.3						
Pleiocarpa pycnantha	5	31	6.5	2.4 ± 0.5	8.8	8.8				
Prioria balsamifera	8	38	18.4	1.6 ± 0.6	13.5	9.5 ± 2	sub-annual			
Prioria oxyphylla	10	94	6.4	2 ± 0.8	11.9	1.8	sub-annual		-0.132 (t-3)	
Pycnanthus angolensis	5	43	2.3	2.2						
Quassia silvestris	6	52	1.9	2.2						
Radlkofera calodendron	5	32	3.1	2.2						
Scorodophloeus zenkeri	4	27	11.1	1.8 ± 1.3	11.4					
Staudtia kamerunensis	4	38	2.6	5.4						
Strombosia grandifolia	8	85	0.0							
Strombosia pustulata	5	48	0.0							
Strombosiopsis tetrandra	7	47	2.1	2.2						
Symphonia globulifera	8	70	0.0							
Synsepalum msolo	5	31	6.5	1.6 ± 0.8						
Synsepalum subcordatum	8	41	9.8	1.7 ± 0.6	10.9	9.3				
Tabernaemontana crassa	1	10	0.0							
Tessmannia anomala	3	30	0.0							
Tetrorchidium didymostemon	5	33	3.0	5.4						
Treculia africana	5	44	9.1	1.9 ± 0.8	9.8	3.5 ± 0.5				-0.137 (t-3)
Tricalysia pallens	1	10	0.0							
Trichilia gilgiana	2	13	0.0							
Trichilia prieuriana	4	32	0.0							
Trichilia rubescens	6	43	4.7	2.2 ± 0						
Tridesmostemon omphalocarpoides	5	60	3.3	1.4 ± 0.6	8.2	8.2				
Trilepisium madagascariense	4	44	0.0							
Turraeanthus africanus	7	48	0.0							
Xylopia aethiopica	8	50	8.0	5 ± 6.1	14.4	3.6				
Xylopia cupularis	1	13	0.0							
**deciduous**										
Albizia adianthifolia	7	57	40.4	2 ± 1.2	11	9.8 ± 5.2	sub-annual	-0.189 (t0)	0.238 (t-1)	0.175 (t0)
Albizia ferruginea	10	70	34.3	2.7 ± 1.7	14.3	5.2 ± 3.2	annual		-0.166 (t0)	-0.152 (t-1)
Allophylus africanus	7	38	34.2	2 ± 0.6	12.3	8.4 ± 7.3		-0.155 (t0)	0.172 (t0)	0.172 (t0)
Alstonia boonei	1	1	100.0	1.1						
Amphimas pterocarpoides	9	70	34.3	1.8 ± 0.8	7.1	4 ± 3.7		-0.134 (t-1)	0.197 (t0)	0.257 (t0)
Anthonotha macrophylla	7	63	19.0	2.8 ± 2.2	7.4	7.8	sub-annual	-0.151 (t0)	0.187 (t0)	0.154 (t0)
Antiaris toxicaria	2	7	14.3	2.2 ± 0	7.8	7.8			0.196 (t-1)	0.189 (t0)
Antrocaryon nannanii	5	52	57.7	2.8 ± 1.5	14.3	13.3 ± 2.5			-0.197 (t-2)	-0.141 (t0)
Baphia laurifolia	3	13	7.7	1.1						
Berlinia grandiflora	3	30	13.3	1.9 ± 1	4.2	4.8				
Brachystegia laurentii	3	5	60.0	1.4 ± 0.6	12					
Caloncoba welwitschii	1	8	0.0							
Canarium schweinfurthii	5	40	30.0	2.1 ± 1.6	21.9	12.3 ± 5.1	sub-annual			
Celtis tessmannii	6	40	0.0							
Cola lateritia	7	57	8.8	2 ± 0.5	14.1	11.3				
Combretum lokele	1	7	14.3	3.2						
Copaifera mildbraedii	4	26	19.2	1.3 ± 0.5	15	16.5				
Dialium excelsum	11	76	25.0	1.8 ± 1	12.4	8 ± 2.6	annual			-0.127 (t-3)
Discoglypremna caloneura	2	18	61.1	2.5 ± 1.1	6.4	5.6 ± 1.7		-0.186 (t0)	0.169 (t0)	0.19 (t0)
Entandrophragma angolense	5	38	47.4	1.5 ± 0.5	8.7	4.3 ± 2			-0.223 (t-2)	-0.19 (t-1)
Entandrophragma candollei	8	50	32.0	2.2 ± 1.1	16.5	12.3 ± 0.9				
Entandrophragma cylindricum	9	84	28.6	1.8 ± 0.8	10.9	10.6 ± 6.3		-0.179 (t0)		
Erythrophleum suaveolens	5	40	50.0	4 ± 2.5	6.7	5.9 ± 2.2	annual	0.197 (t-2)	-0.194 (t-1)	-0.188 (t0)
Fernandoa adolfi-friderici	7	61	41.0	2 ± 1.5	8	4 ± 3.9	annual	-0.251 (t0)	0.281 (t0)	0.214 (t0)
Greenwayodendron suaveolens	2	18	5.6	1.1						
Irvingia grandifolia	7	70	44.3	3.5 ± 5.3	11.4	9.9 ± 2.1			-0.155 (t0)	-0.138 (t0)
Klainedoxa gabonensis	8	71	4.2	2.8 ± 1.5	7.9	7.9 ± NA			-0.139 (t-1)	
Lasiodiscus fasciculiflorus	1	5	0.0							
Milicia excelsa	11	97	56.7	2.6 ± 1.6	10.3	7.1 ± 3.6	annual	-0.159 (t-1)	0.245 (t0)	0.213 (t0)
Monodora myristica	3	21	14.3	1.1 ± 0	0.9					
Nauclea diderrichii	7	49	6.1	1.8 ± 0.6	4.5					
Parkia bicolor	3	15	46.7	1.9 ± 1.2	11.8	10.9 ± 6				-0.138 (t-1)
Pentaclethra macrophylla	5	46	23.9	1.7 ± 0.8	14	12.7 ± 6.3		0.132 (t0)		
Pericopsis elata	5	50	40.0	2 ± 0.8	3.5	1.8 ± 0.6	annual	-0.128 (t0)	0.26 (t0)	0.347 (t0)
Petersianthus macrocarpus	3	26	3.8	2.2						
Pteleopsis hylodendron	8	36	36.1	2.3 ± 0.9	9	8.3 ± 6.3	sub-annual		-0.175 (t-3)	-0.143 (t-2)
Pterocarpus soyauxii	6	45	75.6	2 ± 1.4	20.2	14.1 ± 1.9				
Pterygota bequaertii	8	73	26.0	1.8 ± 1.1	10.6	9.8 ± 1				-0.156 (t-2)
Ricinodendron heudelotii	7	60	60.0	4.3 ± 2.4	3.1	2.3 ± 1.4	annual	-0.28 (t0)	0.428 (t0)	0.487 (t0)
Rinorea welwitschii	2	13	0.0							

Table S2 continued

species	nr ind	nr obs years	% years with phase	duration phase (w)	variability (SD)		cyclicity	cross-correlations		
					intra-sp.	inter-ann.		precip	sun hours	tmax
**deciduous**										
Sterculia dawei	5	61	60.7	2.3 ± 1.4	18.5	15.1 ± 1.5	sub-annual			
Vitex congolensis	3	31	29.0	1.4 ± 0.5	2.3	0.9 ± 0.5	annual	-0.229 (t0)	0.19 (t0)	0.278 (t0)
Zanthoxylum gilletii	10	82	7.3	3.6 ± 3.2	8.2				0.137 (t0)	
**unclassified**										
Cleistanthus polystachyus	1	2	0.0							
Colletoecema dewevrei	1	2	0.0							
Dialium pentandrum	3	23	8.7	5.8 ± 6.3	7.8	7.8				
Drypetes leonensis	1	3	0.0							
Irvingia gabonensis	1	13	7.7	1.1						
Macaranga monandra	3	9	22.2	2.5 ± 1.7	4.8					
Newtonia devredii	1	2	0.0							
Syzygium congolense	2	5	0.0							

Table S3: Species-specific characteristics of the phenological cycles of canopy turnover. For each species, the number of individuals observed, the total observation years, the percentage of observation years with observed phenophases and the average duration (± SD) of a phenophase in weeks are indicated. Variability in the timing of phenophases across years is represented as inter-annual SD and within species (across individuals) as intra-species SD. Timing of fourier-based cyclicity is indicated. Significant correlations of time-series cross-correlation analysis between phenological observations and precipitation, amount of sun hours and maximum temperature are provided, with time of lag of the phenological observations in relation to each climatological variable indicated. Time t0 indicates significant in-phase cross-correlations ($p<0.05$); t-x indicates the time-lag in months of significant lagged cross-correlations ($p<0.05$).

species	nr ind	nr obs years	% years with phase	duration phase (w)	variability (SD)		cyclicity	cross-correlations		
					intra-sp.	inter-ann.		precip	sun hours	tmax
**evergreen**										
Afrostyrax lepidophyllus	5	27	11.1	2.2 ± 0	10.9					
Allanblackia floribunda	4	28	7.1	5.4 ± 4.6						
Angylocalyx pynaertii	7	41	12.2	2.4 ± 0.5	8.6				-0.15 (t-1)	
Anonidium mannii	3	31	0.0							
Baikiaea insignis	8	34	0.0							
Baphia pubescens	4	15	26.7	5.1 ± 4.1	2.8	2.8				
Barteria fistulosa	1	3	33.3	1.8 ± 0.6	4.5	4.5				
Beilschmiedia louisii	4	42	7.1	4 ± 4.1	4.8	4.8				
Blighia welwitschii	8	58	15.5	3.6 ± 2.4	10.9	9.8 ± 5.4				-0.138 (t-2)
Carapa procera	9	56	5.4	9.1 ± 8.7	12.7				-0.151 (t0)	
Celtis mildbraedii	9	57	7.0	2.2 ± 0.9	11.4	13.8				
Chlamydocola chlamydantha	3	11	0.0							
Chrysophyllum africanum	4	33	12.1	4.3 ± 3.4	8.5	4.4				
Chrysophyllum lacourtianum	4	48	4.2	6 ± 0.8						
Chrysophyllum pruniforme	2	17	0.0							
Coelocaryon preussii	5	48	6.3	9.8 ± 7.6	2.4					
Cola griseiflora	8	62	3.2	3.8 ± 0.8						
Croton mubango	1	13	0.0							
Cynometra alexandri	2	2	0.0							
Cynometra hankei	5	30	0.0							
Dacryodes edulis	4	27	0.0							
Dacryodes osika	4	43	25.6	3 ± 1.8	7.6	6.3 ± 2.7	annual		0.161 (t0)	0.245 (t0)
Dactyladenia dewevrei	3	12	0.0							
Desplatsia dewevrei	6	36	0.0							
Dialium corbisieri	1	7	0.0							
Dialium pachyphyllum	7	47	0.0							
Dichostemma glaucescens	4	18	0.0							
Diogoa zenkeri	3	9	0.0							
Diospyros crassiflora	4	38	2.6	2.2						
Diospyros hoyleana	7	50	2.0	40.1						
Diospyros iturensis	9	84	3.6	4.3 ± 2.2	9.7	9.7				
Drypetes gossweileri	5	29	3.4	3.2						
Drypetes spinosodentata	4	28	0.0							
Eriocoelum microspermum	5	40	5.0	1.6 ± 0.8						
Ficus ardisioides	1	5	0.0							
Funtumia africana	1	1	0.0							
Garcinia epunctata	2	19	5.3	2.2						
Garcinia punctata	5	37	0.0							
Garcinia smeathmanii	7	36	0.0							
Gilbertiodendron dewevrei	8	45	2.2	2.2						
Gilletiodendron mildbraedii	5	38	10.5	3.1 ± 1.6	9.5	4.3				
Guarea cedrata	1	8	12.5	2.2						
Hymenocardia ulmoides	7	34	14.7	3 ± 0.9	2.7	1.2		-0.204 (t-1)	0.163 (t0)	0.22 (t0)
Isolona hexaloba	7	84	1.2	2.2						
Leplaea laurentii	5	53	1.9	2.2						
Macaranga barteri	3	21	9.5	2.7 ± 0.8						
Mammea africana	11	64	4.7	2.2 ± 0.7	13	11.8				-0.143 (t-1)
Maranthes glabra	2	10	10.0	7.6						
Massularia acuminata	2	5	0.0							
Microdesmis puberula	4	33	0.0							
Myrianthus arboreus	5	18	0.0							
Napoleonaea vogelii	4	36	5.6	1.6 ± 0.8						
Omphalocarpum ghesquierei	1	13	15.4	3.2 ± 1.5						
Ongokea gore	6	66	1.5	2.2						
Pancovia harmsiana	2	19	10.5	13 ± 16.9						
Pancovia laurentii	7	62	8.1	3.1 ± 2.3	5.9			-0.139 (t-2)	0.132 (t0)	0.184 (t0)

Table S3 continued

species	nr ind	nr obs years	% years with phase	duration phase (w)	variability (SD)		cyclicity	cross-correlations		
					intra-sp.	inter-ann.		precip	sun hours	tmax
**evergreen**										
Panda oleosa	5	61	1.6	2.2						
Paramacrolobium coeruleum	2	20	5.0	3.2						
Parinari excelsa	1	5	0.0							
Pausinystalia macroceras	4	31	9.7	1.8 ± 1.3	9.1					
Phyllocosmus africanus	9	45	28.9	3.7 ± 2.7	20.4	11 ± 0.6				
Platysepalum chevalieri	3	19	10.5	3.2 ± 1.5						
Pleiocarpa pycnantha	5	31	9.7	5.8 ± 4.8	10.8	13.2				
Prioria balsamifera	8	38	39.5	3.6 ± 2.1	9.4	7.1 ± 3.1			-0.179 (t-2)	-0.199 (t-2)
Prioria oxyphylla	10	94	12.8	4 ± 4	9	2.2	supra-annual			
Pycnanthus angolensis	5	43	0.0							
Quassia silvestris	6	52	5.8	3.2 ± 3.1	6.2	4.1			0.144 (t-1)	0.158 (t0)
Radlkofera calodendron	5	32	9.4	2.5 ± 0.6	11.1	11.1				
Scorodophloeus zenkeri	4	27	3.7	4.3						
Staudtia kamerunensis	4	38	0.0							
Strombosia grandifolia	8	85	1.2	1.1						
Strombosia pustulata	5	48	4.2	2.7 ± 0.8						
Strombosiopsis tetrandra	7	47	2.1	3.2						
Symphonia globulifera	8	70	2.9	4.3 ± 0						
Synsepalum msolo	5	31	6.5	1.6 ± 0.8						
Synsepalum subcordatum	8	41	14.6	2.7 ± 1.2	12.6	10.4				
Tabernaemontana crassa	1	10	0.0							
Tessmannia anomala	3	30	10.0	2.6 ± 1.8	7.4	3.7		-0.17 (t-2)	0.156 (t-1)	0.169 (t0)
Tetrorchidium didymostemon	5	33	9.1	5.1 ± 4.1	7.4					
Treculia africana	5	44	25.0	3.2 ± 1.8	8.2	2.2			-0.183 (t-3)	-0.2 (t-3)
Tricalysia pallens	1	10	0.0							
Trichilia gilgiana	2	13	0.0							
Trichilia prieuriana	4	32	0.0							
Trichilia rubescens	6	43	0.0							
Tridesmostemon omphalocarpoides	5	60	0.0							
Trilepisium madagascariense	4	44	0.0							
Turraeanthus africanus	7	48	4.2	6.5 ± 4.6						
Xylopia aethiopica	8	50	4.0	4.6 ± 2.8	11.1					
Xylopia cupularis	1	13	0.0							
**deciduous**										
Albizia adianthifolia	7	57	47.4	3.8 ± 2.5	12.5	10.9 ± 3.7		-0.185 (t0)	0.193 (t0)	0.2 (t0)
Albizia ferruginea	10	70	65.7	4.6 ± 2.8	14.3	9.4 ± 5.2			-0.184 (t-1)	-0.15 (t0)
Allophylus africanus	7	38	50.0	3.4 ± 1.7	10.5	8.5 ± 2.1	sub-annual	-0.215 (t0)	0.235 (t0)	0.228 (t0)
Alstonia boonei	1	1	100.0	1.1						
Amphimas pterocarpoides	9	70	65.7	4.6 ± 4	6.6	5.8 ± 1.6	annual	-0.231 (t0)	0.322 (t0)	0.179 (t0)
Anthonotha macrophylla	7	63	31.7	3 ± 1.4	9.9	7 ± 4.4	annual	-0.16 (t-2)	0.128 (t0)	0.157 (t0)
Antiaris toxicaria	2	7	85.7	3.2 ± 1.7	9.6	10.2			0.181 (t-1)	
Antrocaryon nannanii	5	52	50.0	3.1 ± 1.2	15.9	13.5 ± 2.4	sub-annual			
Baphia laurifolia	3	13	7.7	2.2						
Berlinia grandiflora	3	30	3.3	4.3						
Brachystegia laurentii	3	5	40.0	2.2 ± 1.5						
Caloncoba welwitschii	1	8	0.0							
Canarium schweinfurthii	5	40	50.0	3.9 ± 2.5	12	4.9 ± 1.6				-0.215 (t-2)
Celtis tessmannii	6	40	7.5	5.1 ± 4.1	10.5					
Cola lateritia	7	57	43.9	3.5 ± 2.5	10.7	8.1 ± 4.1	annual	-0.131 (t-1)	-0.136 (t-3)	-0.184 (t-2)
Combretum lokele	1	7	85.7	2.9 ± 0.9	4.3	4.3		-0.199 (t0)	0.288 (t0)	-0.186 (t-3)
Copaifera mildbraedii	4	26	46.2	3.7 ± 2.3	7.7	7.4 ± 3.8	annual	-0.147 (t0)	0.13 (t0)	-0.15 (t-3)
Dialium excelsum	11	76	50.0	3.3 ± 2.1	9.8	7.5 ± 4.4	sub-annual	-0.152 (t0)	0.152 (t0)	0.234 (t0)
Discoglypremna caloneura	2	18	38.9	2.9 ± 1.9	6.1	5.9 ± 5.5		-0.161 (t0)	0.184 (t0)	0.144 (t0)
Entandrophragma angolense	5	38	52.6	2.5 ± 1	8.9	5.2 ± 5.8	annual		-0.181 (t0)	-0.183 (t0)
Entandrophragma candollei	8	50	66.0	4.1 ± 1.8	16.3	14.2 ± 5.9	sub-annual			
Entandrophragma cylindricum	9	84	51.2	3.8 ± 2.1	8.8	5.9 ± 4	sub-annual	-0.252 (t0)	0.282 (t0)	0.229 (t0)
Erythrophleum suaveolens	5	40	20.0	3 ± 0.8	3.4	3.4	annual	-0.263 (t0)	0.32 (t0)	0.259 (t0)
Fernandoa adolfi-friderici	7	61	36.1	3.5 ± 2.3	6.4	3.7 ± 2	annual	-0.143 (t0)	0.21 (t0)	0.198 (t0)
Greenwayodendron suaveolens	2	18	11.1	14.6 ± 17.6						
Irvingia grandifolia	7	70	54.3	4.4 ± 2.6	10.9	9.1 ± 2.4	annual	-0.217 (t-1)	0.24 (t0)	0.261 (t0)
Klainedoxa gabonensis	8	71	4.2	3.1 ± 2.3	14.9	17.6				
Lasiodiscus fasciculiflorus	1	5	0.0							
Milicia excelsa	11	97	53.6	3.9 ± 3.2	9.5	6.1 ± 4.6	annual	-0.216 (t0)	0.222 (t0)	-0.206 (t-1)
Monodora myristica	3	21	47.6	3.2 ± 1.4	1.6	1.1 ± 0.1	annual	-0.281 (t0)	0.37 (t0)	0.418 (t0)
Nauclea diderrichii	7	49	0.0							
Parkia bicolor	3	15	40.0	2.8 ± 1.4	8.6	4.7			-0.165 (t-2)	-0.155 (t-3)
Pentaclethra macrophylla	5	46	17.4	2.9 ± 1.5	9.6	6.5 ± 5.4				
Pericopsis elata	5	50	34.0	4.2 ± 3.5	7	4 ± 1.9	annual	-0.133 (t0)	0.196 (t0)	-0.129 (t-2)
Petersianthus macrocarpus	3	26	19.2	3.4 ± 1.4	10.1	10.1	sub-annual	-0.159 (t-1)	0.192 (t-1)	0.219 (t0)
Pteleopsis hylodendron	8	36	30.6	3.7 ± 1.6	13.1	5.9 ± 3.2			-0.151 (t-3)	-0.154 (t-1)
Pterocarpus soyauxii	6	45	40.0	2.7 ± 1.7	14.4	11.6 ± 2.9			-0.155 (t-3)	
Pterygota bequaertii	8	73	49.3	3.4 ± 3.8	14.3	10.2 ± 4		-0.144 (t0)		
Ricinodendron heudelotii	7	60	46.7	4 ± 2.5	3	2.8 ± 0.8	annual	-0.301 (t0)	0.402 (t0)	0.247 (t0)

Table S3 continued

species	nr ind	nr obs years	% years with phase	duration phase (w)	variability (SD)		cyclicity	cross-correlations		
					intra-sp.	inter-ann.		precip	sun hours	tmax
**deciduous**										
Rinorea welwitschii	2	13	0.0							
Sterculia dawei	5	61	52.5	3 ± 1.1	20.9	14.4 ± 2.5				
Vitex congolensis	3	31	51.6	2.6 ± 1.5	2.2	1.8 ± 0.7	annual	-0.232 (t0)	0.331 (t0)	0.351 (t0)
Zanthoxylum gilletii	10	82	18.3	2.9 ± 1.1	12.5	11.5 ± 7.5				-0.134 (t-3)
**unclassified**										
Cleistanthus polystachyus	1	2	50.0	2.2						
Colletoecema dewevrei	1	2	0.0							
Dialium pentandrum	3	23	17.4	2.4 ± 1	14	9.5				
Drypetes leonensis	1	3	33.3	1.1						
Irvingia gabonensis	1	13	38.5	4.3 ± 2.7	10.6	10.6				
Macaranga monandra	3	9	33.3	4.3 ± 2.5	12.9	8.3				
Newtonia devredii	1	2	0.0							
Syzygium congolense	2	5	40.0	3.2 ± 1.5						

Figure S1: Overview of annual leaf phenological cycles for (a) evergreen, (b) deciduous, and (c) unclassified species. The left column shows the frequency of observations of leaf senescence (black) and turnover (grey) across the individuals studied for each species for the entire observation period from 1937 – 1956. Species-specific observation periods do vary within this full range, with varying start and end years and few missing years. The right column shows the average annual pattern for each species across its individuals with the annual frequency indicated using a color scale. Senescence is indicated in the outer circle, turnover in the inner circle. Note the page-specific color scale for the selected evergreen and deciduous species, with a varying maximum frequency of observations. Grey areas indicate the average timing of the two dry seasons (monthly precipitation < 150 mm). For full list of species including those showing no leaf phenophases, see Table S1.

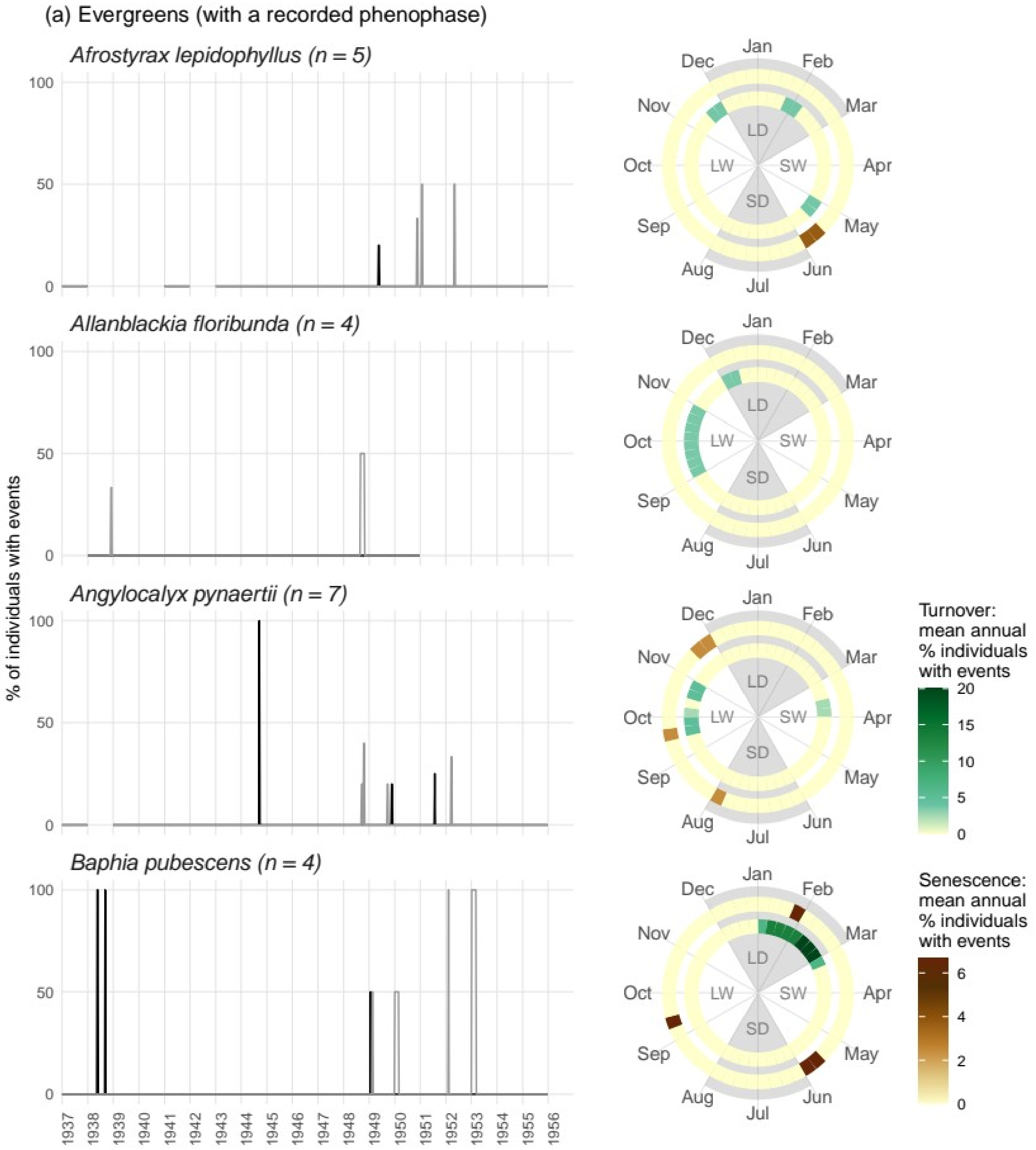

(a) Evergreens – continued

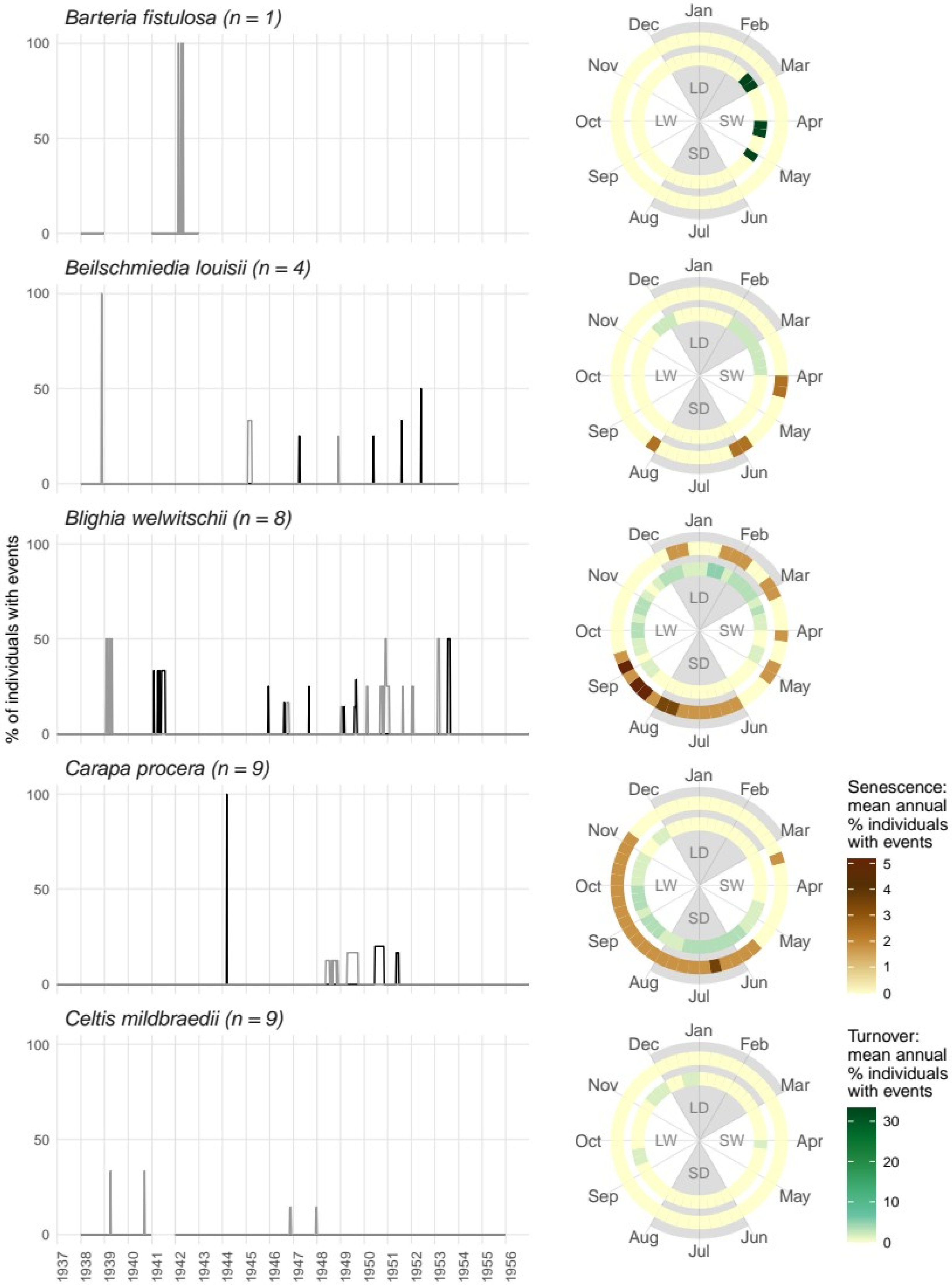

(a) Evergreens – continued

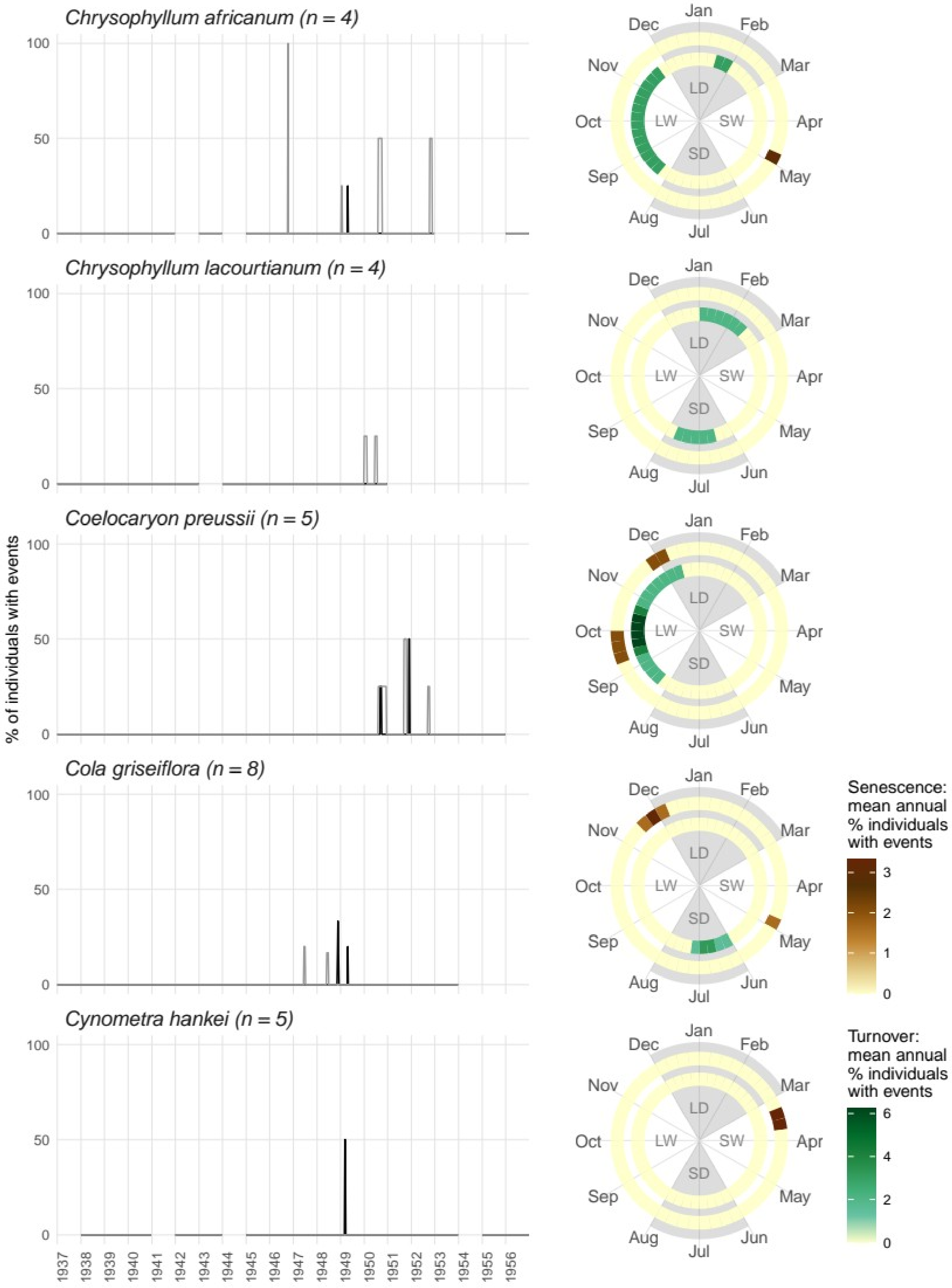

(a) Evergreens – continued

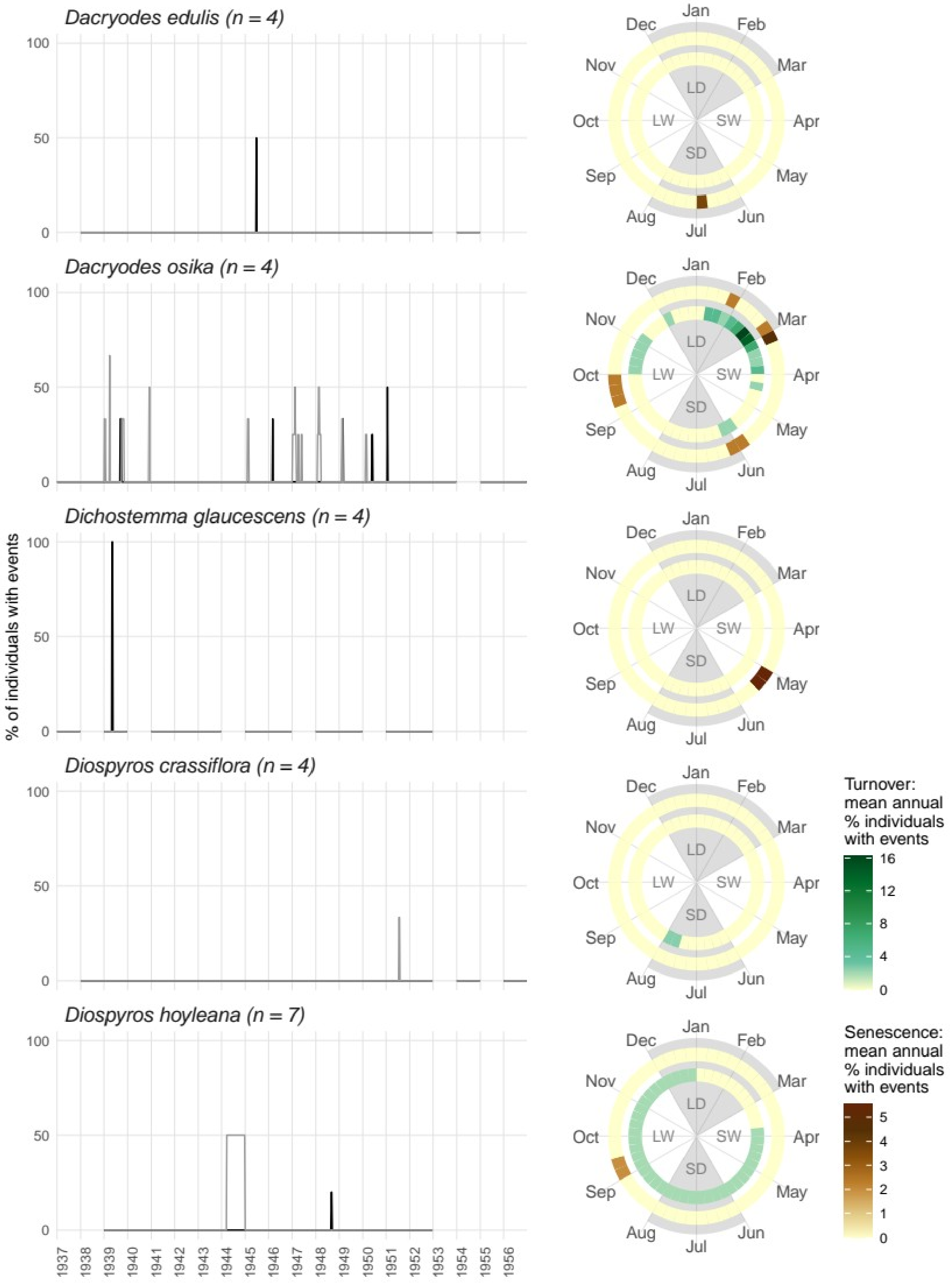

(a) Evergreens – continued

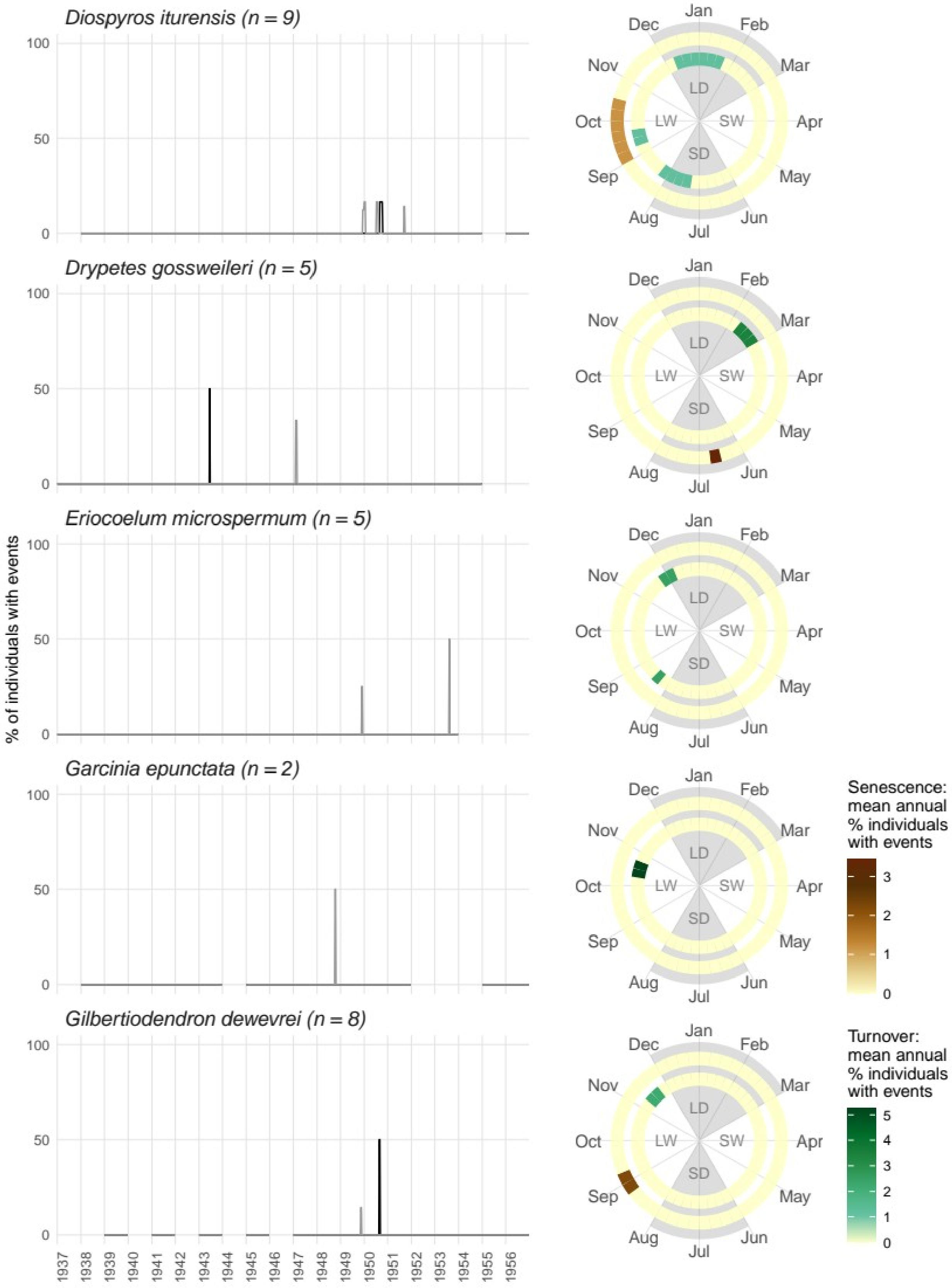

(a) Evergreens – continued

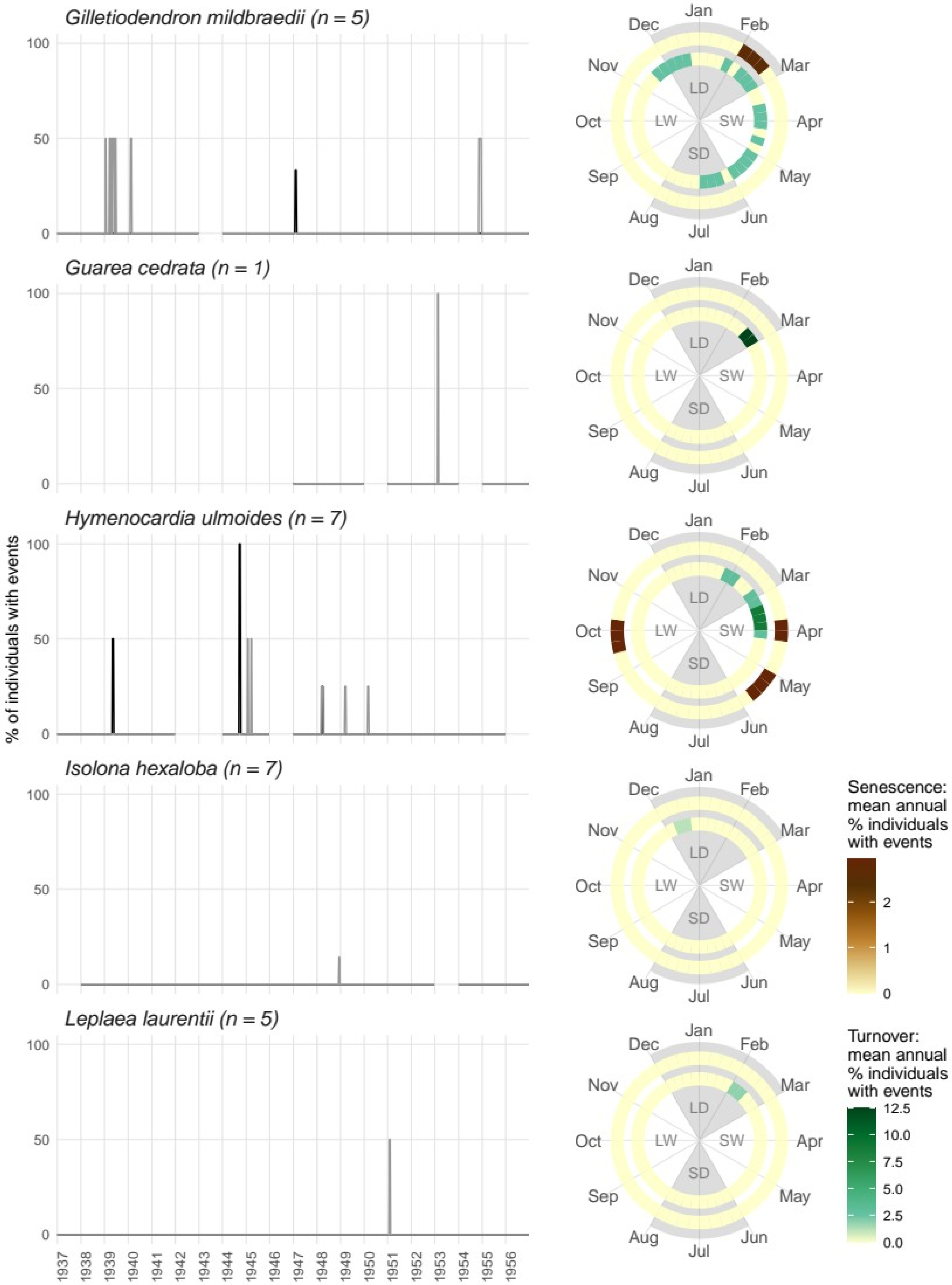

(a) Evergreens – continued

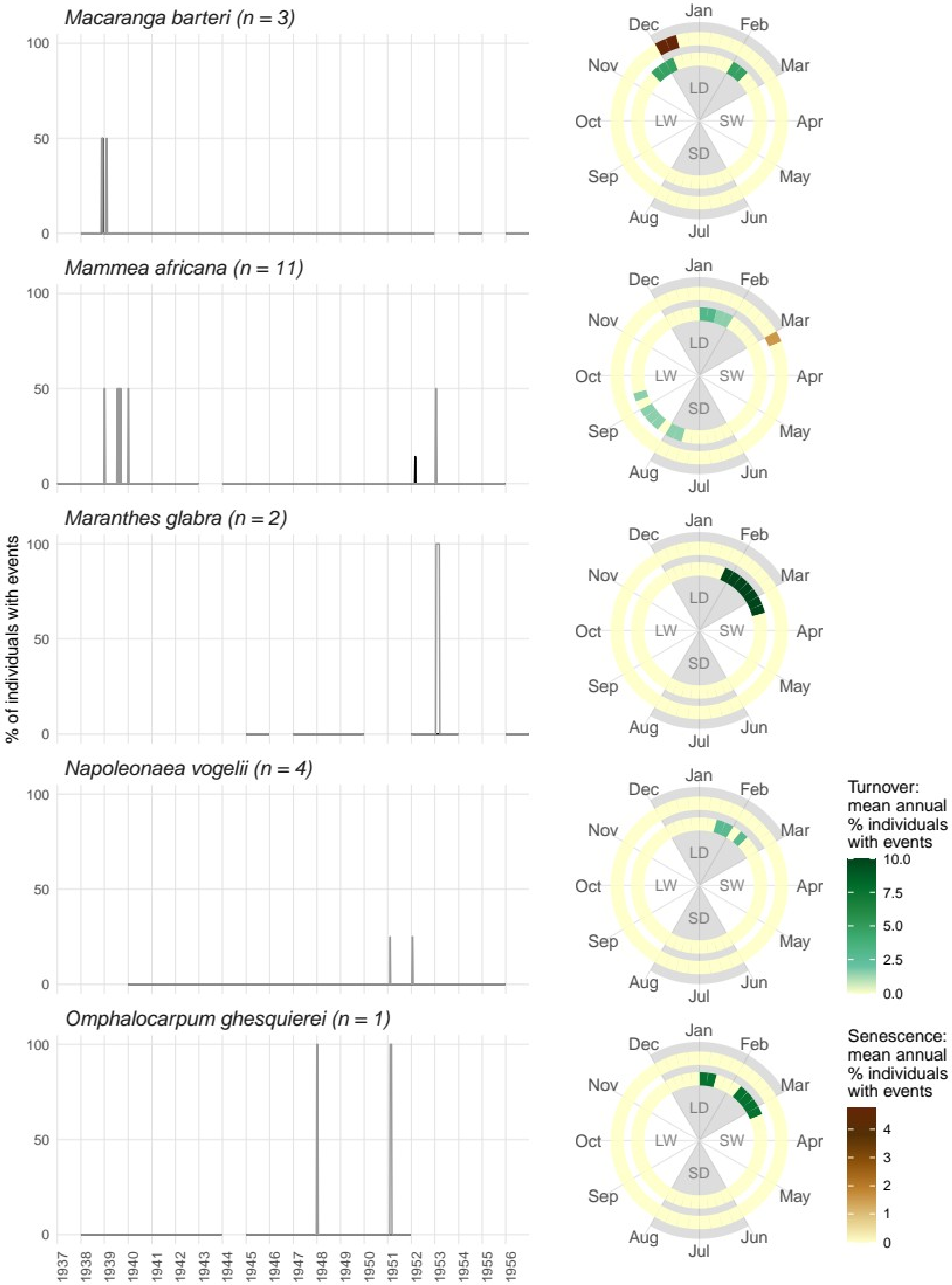

(a) Evergreens – continued

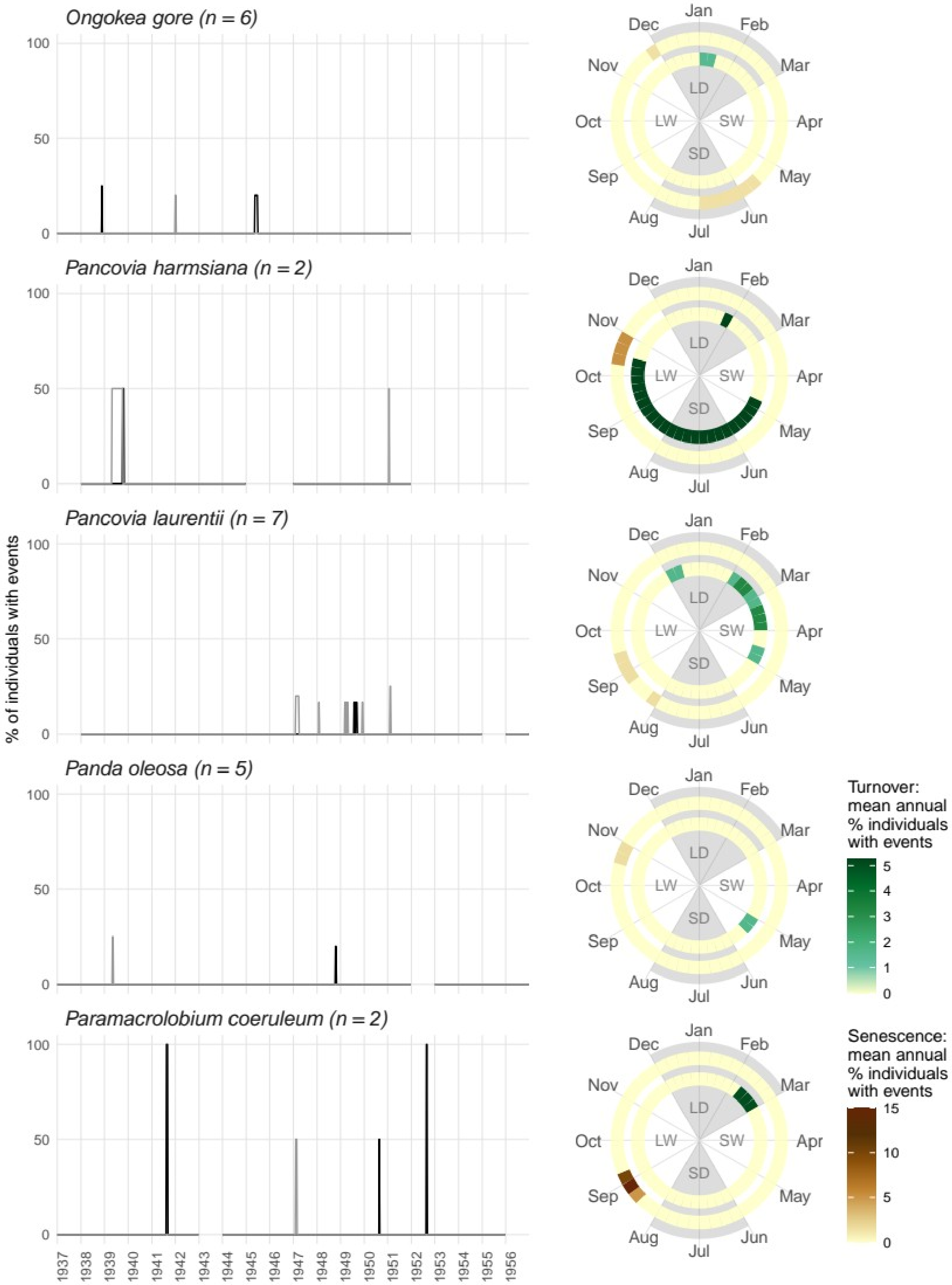

(a) Evergreens – continued

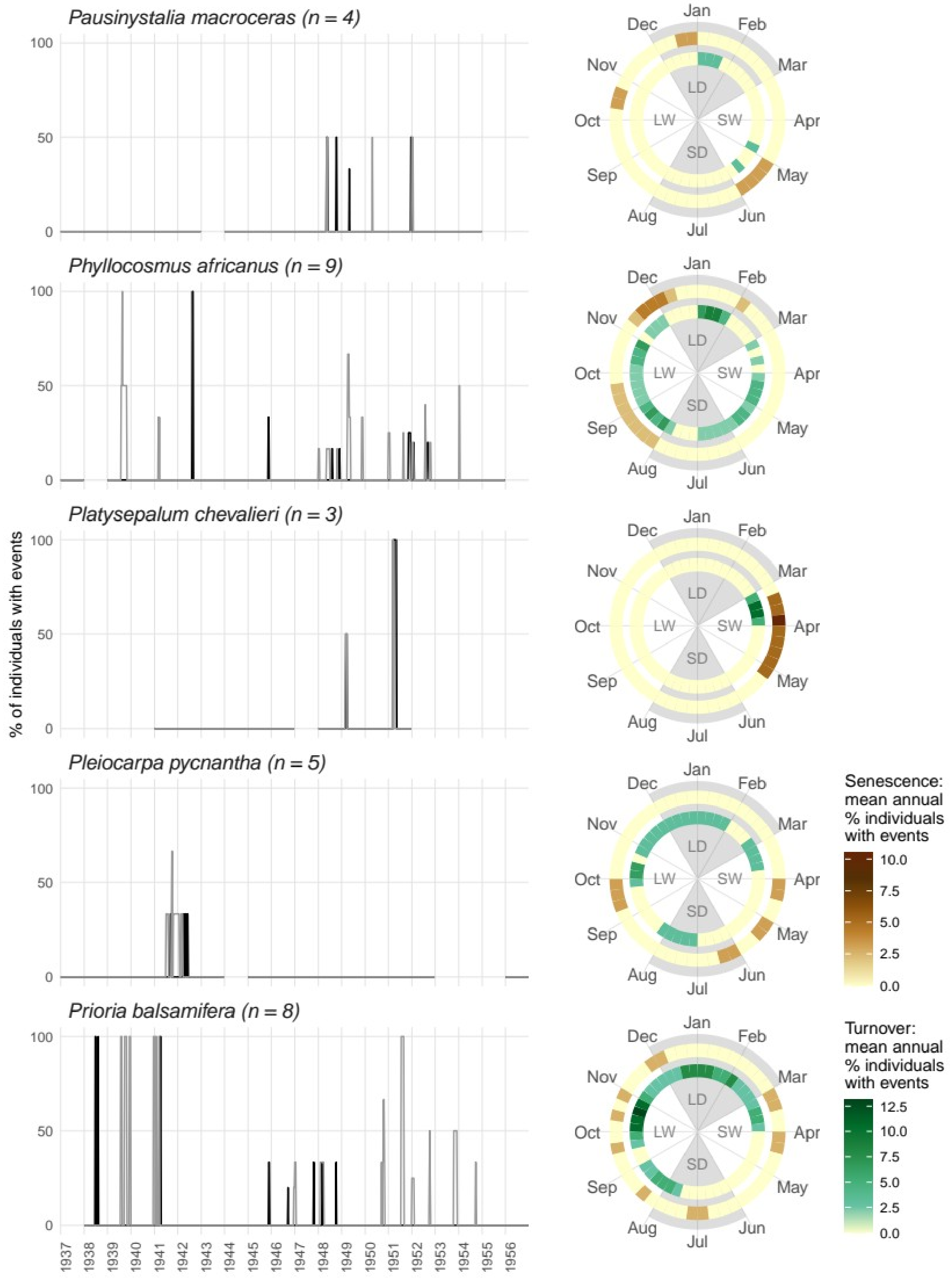

(a) Evergreens – continued

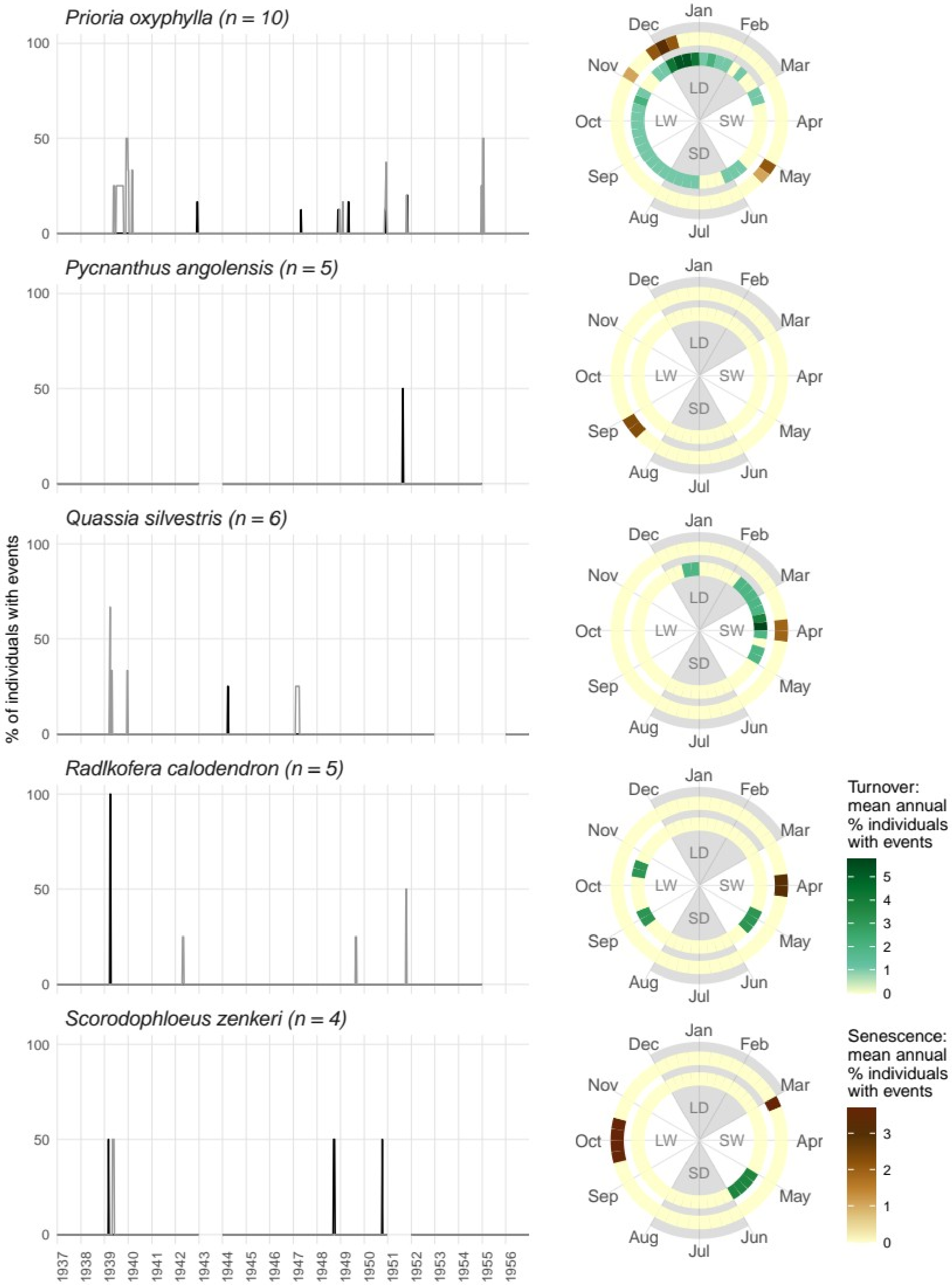

(a) Evergreens – continued

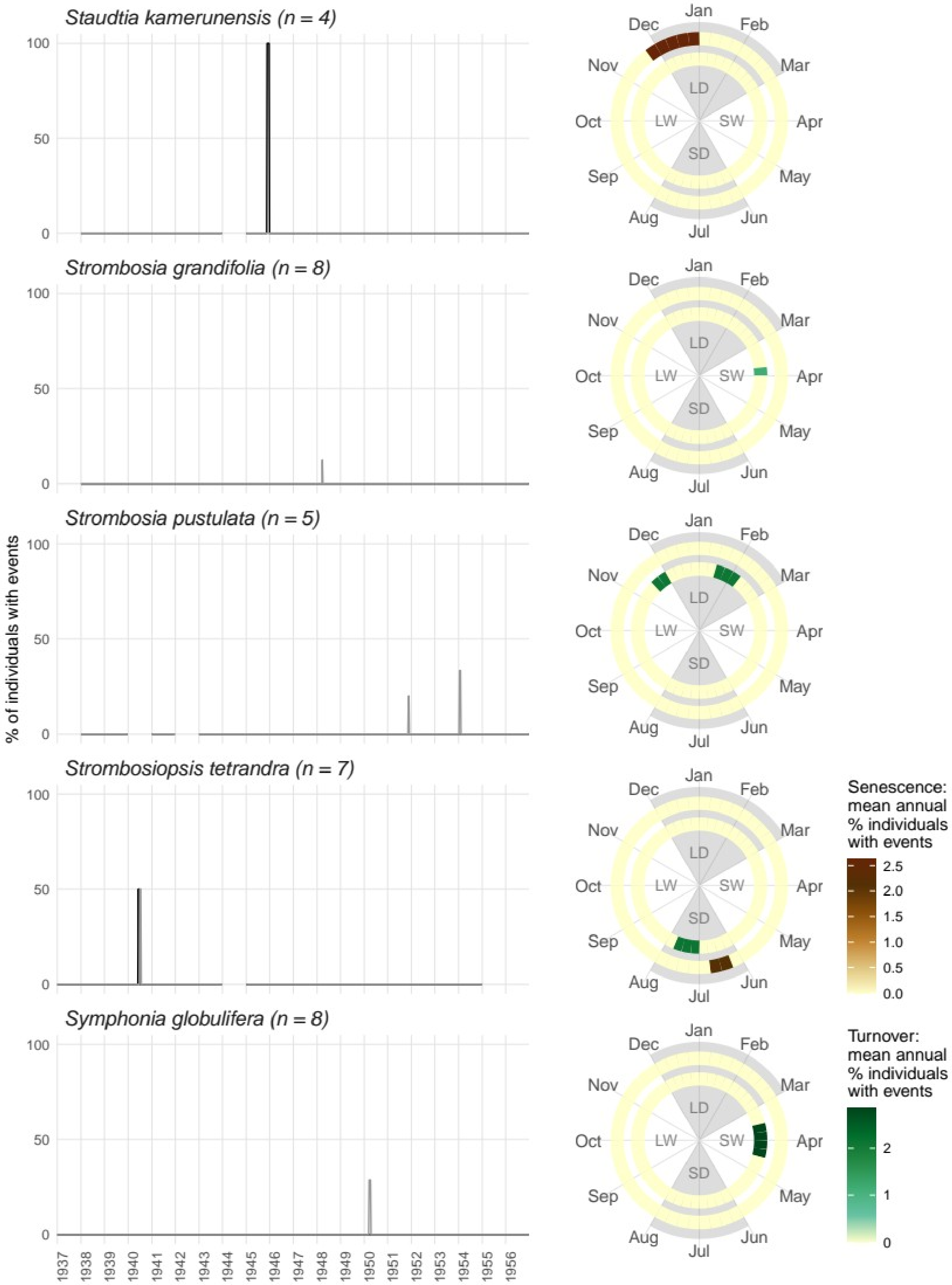

(a) Evergreens – continued

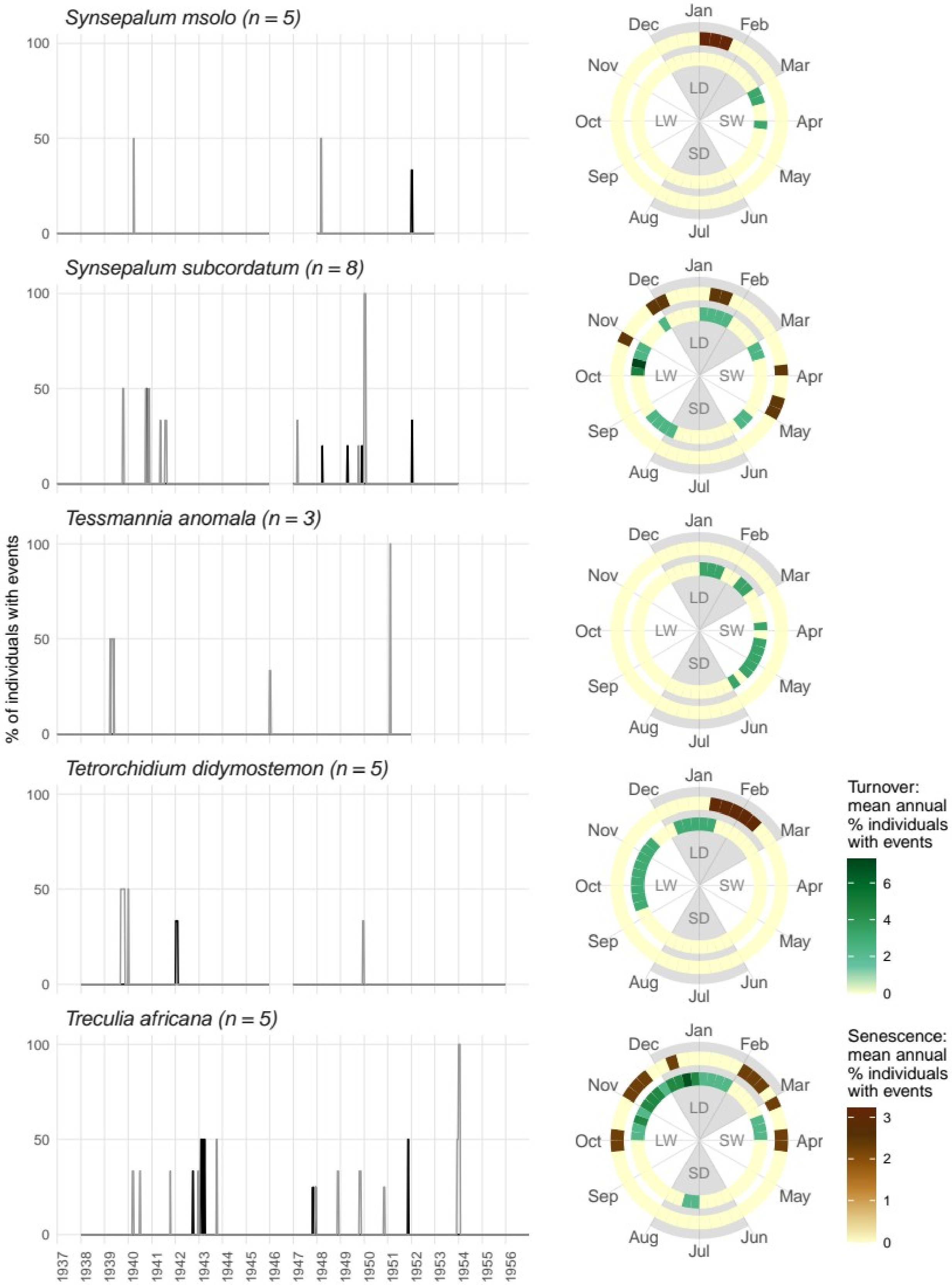

(a) Evergreens – continued

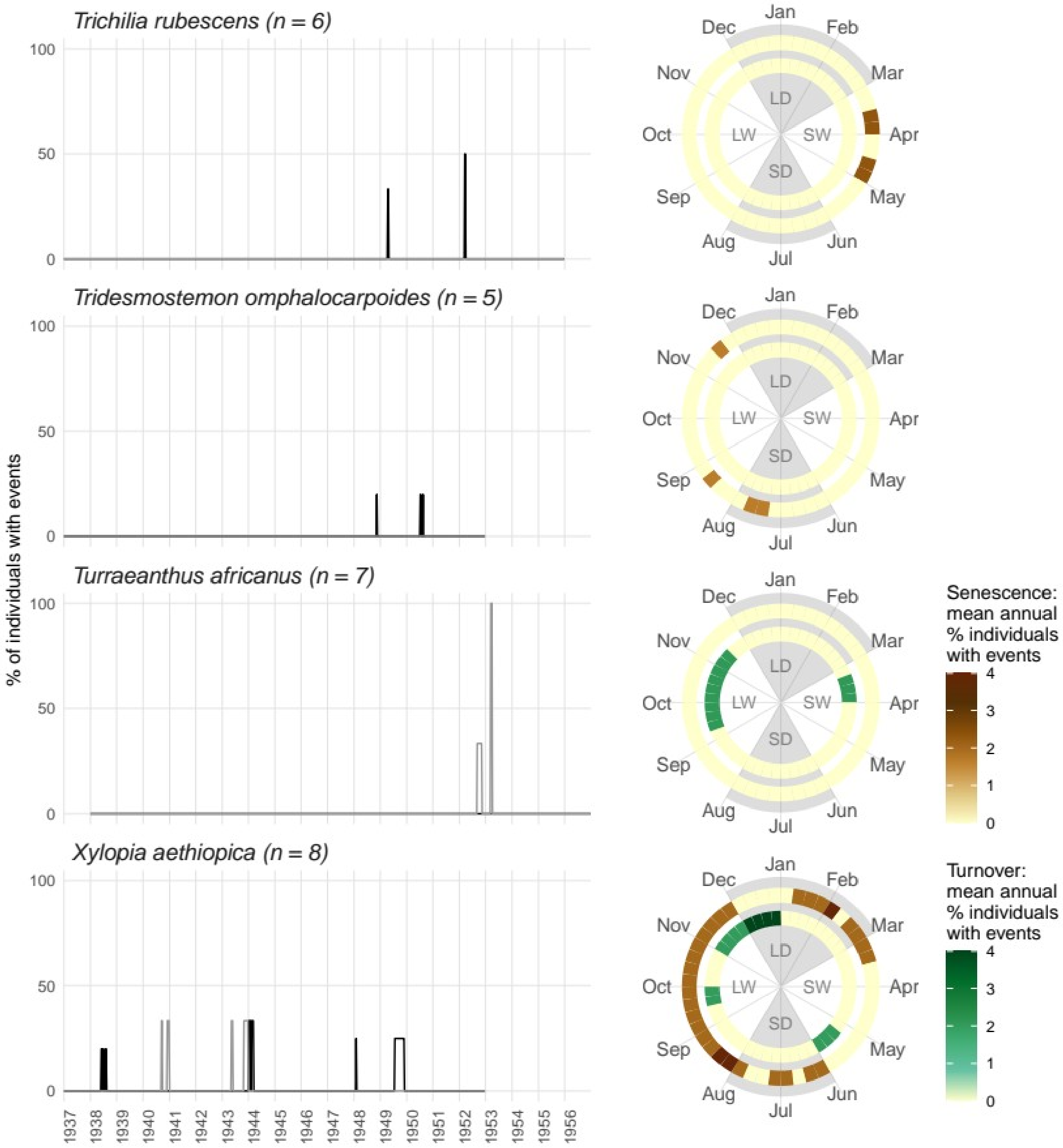

(b) Deciduous

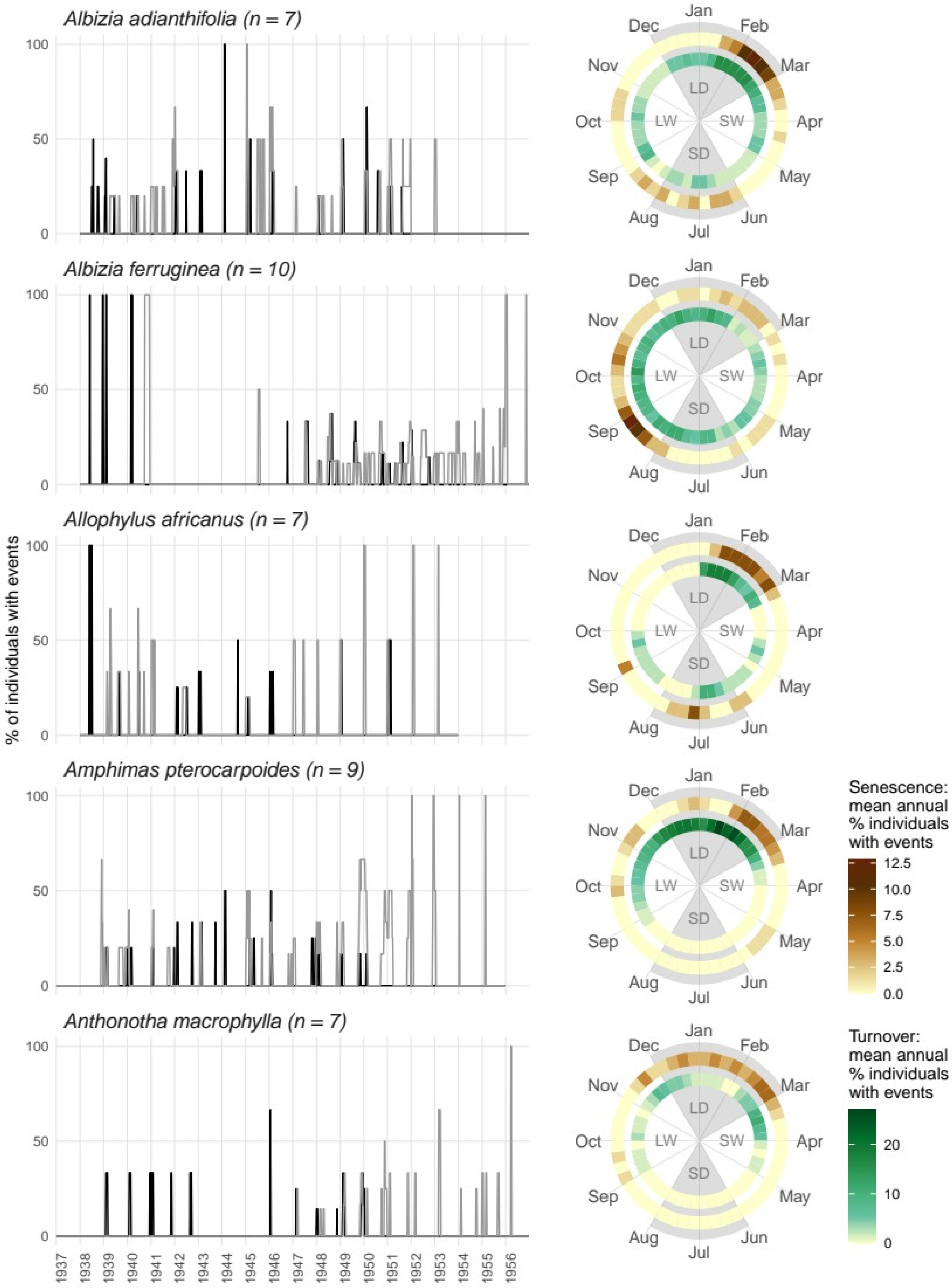

Albizia adianthifolia (n = 7)
Albizia ferruginea (n = 10)
Allophylus africanus (n = 7)
Amphimas pterocarpoides (n = 9)
Anthonotha macrophylla (n = 7)
% of individuals with events
100
50
0
1937
1938
1939
1940
1941
1942
1943
1944
1945
1946
1947
1948
1949
1950
1951
1952
1953
1954
1955
1956
Jan
Feb
Mar
Apr
May
Jun
Jul
Aug
Sep
Oct
Nov
Dec
LD
SW
SD
LW
Senescence: mean annual % individuals with events
12.5
10.0
7.5
5.0
2.5
0.0
Turnover: mean annual % individuals with events
20
10
0

(b) Deciduous – continued

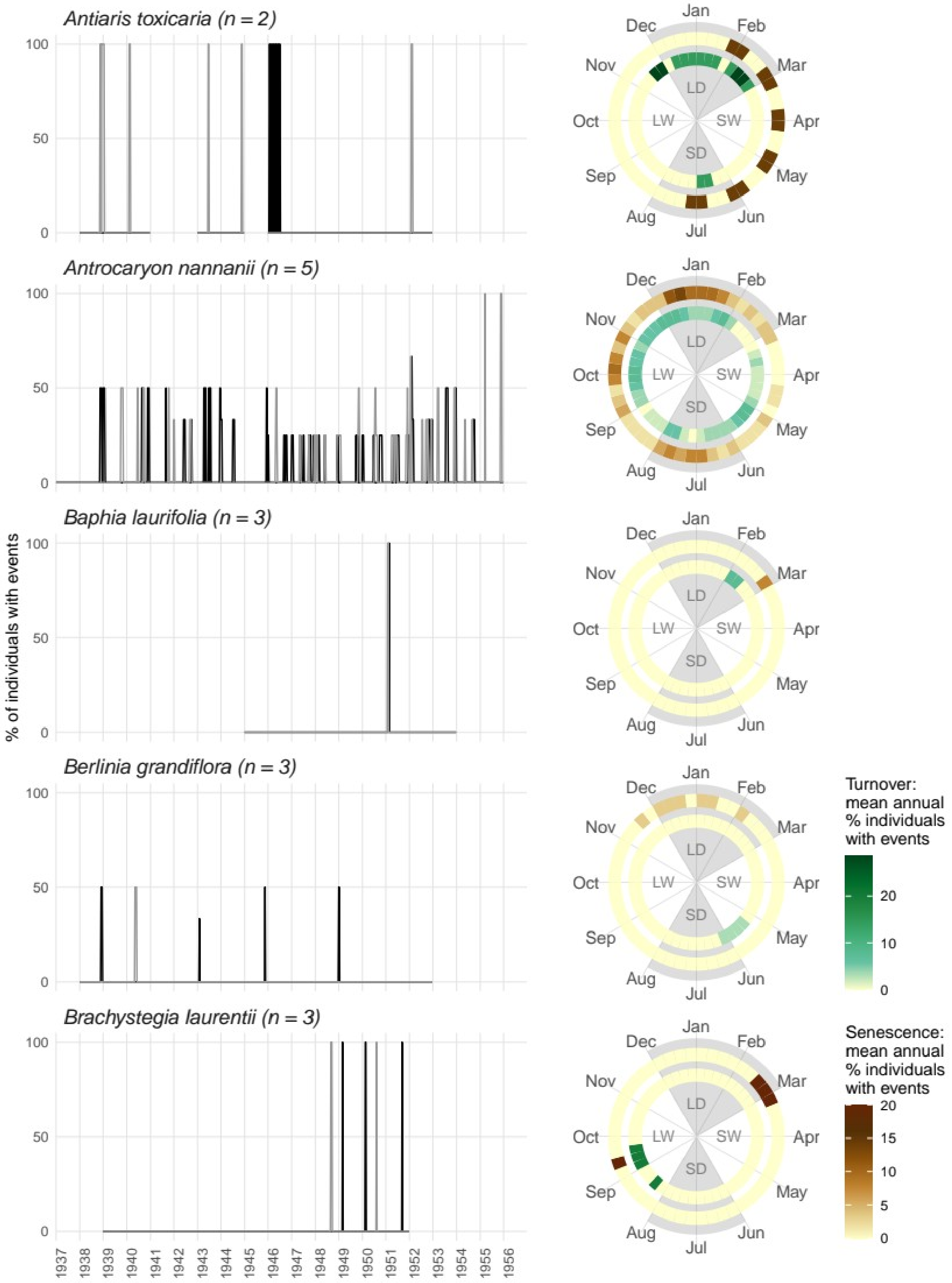

(b) Deciduous – continued

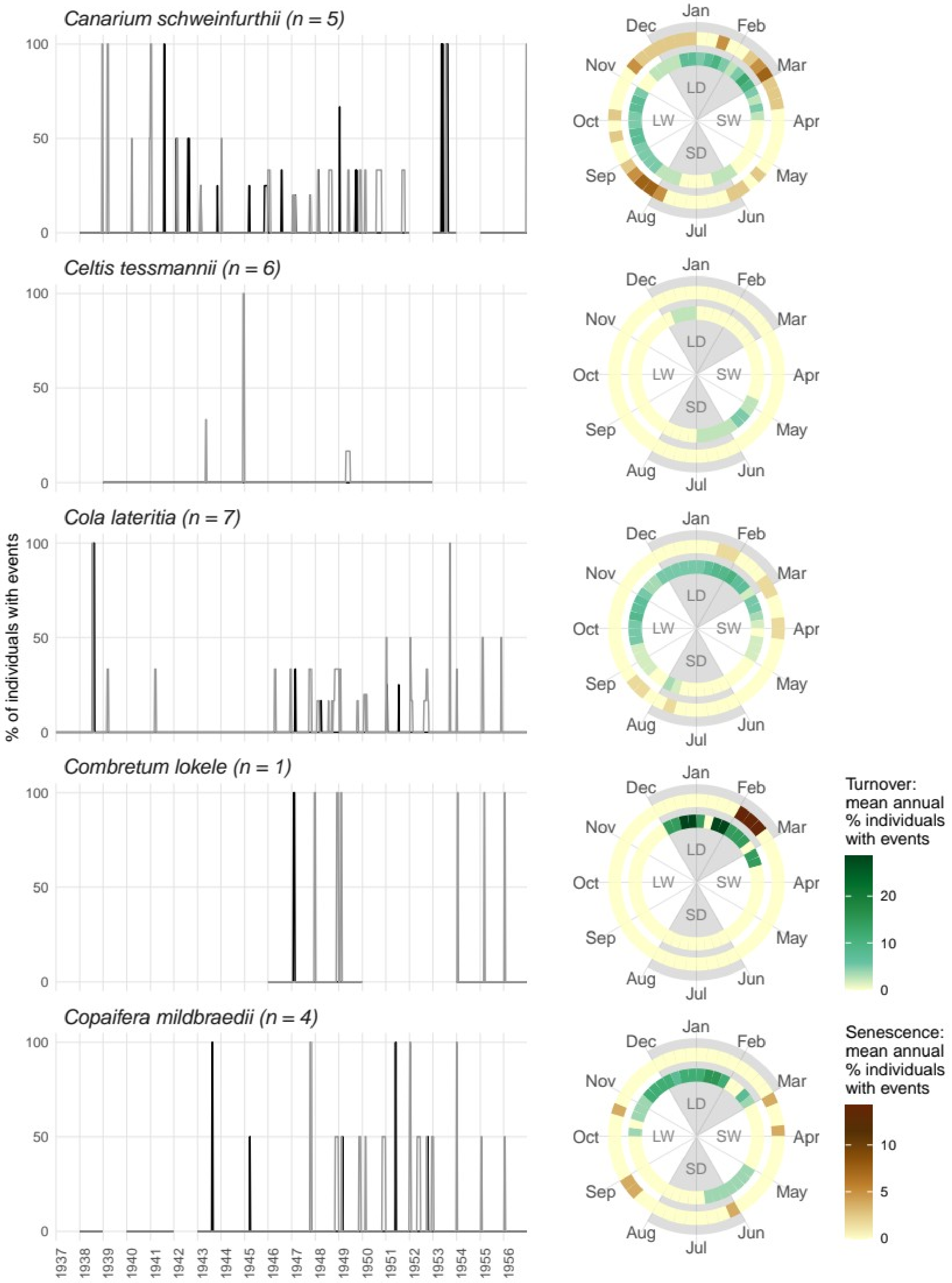

(b) Deciduous – continued

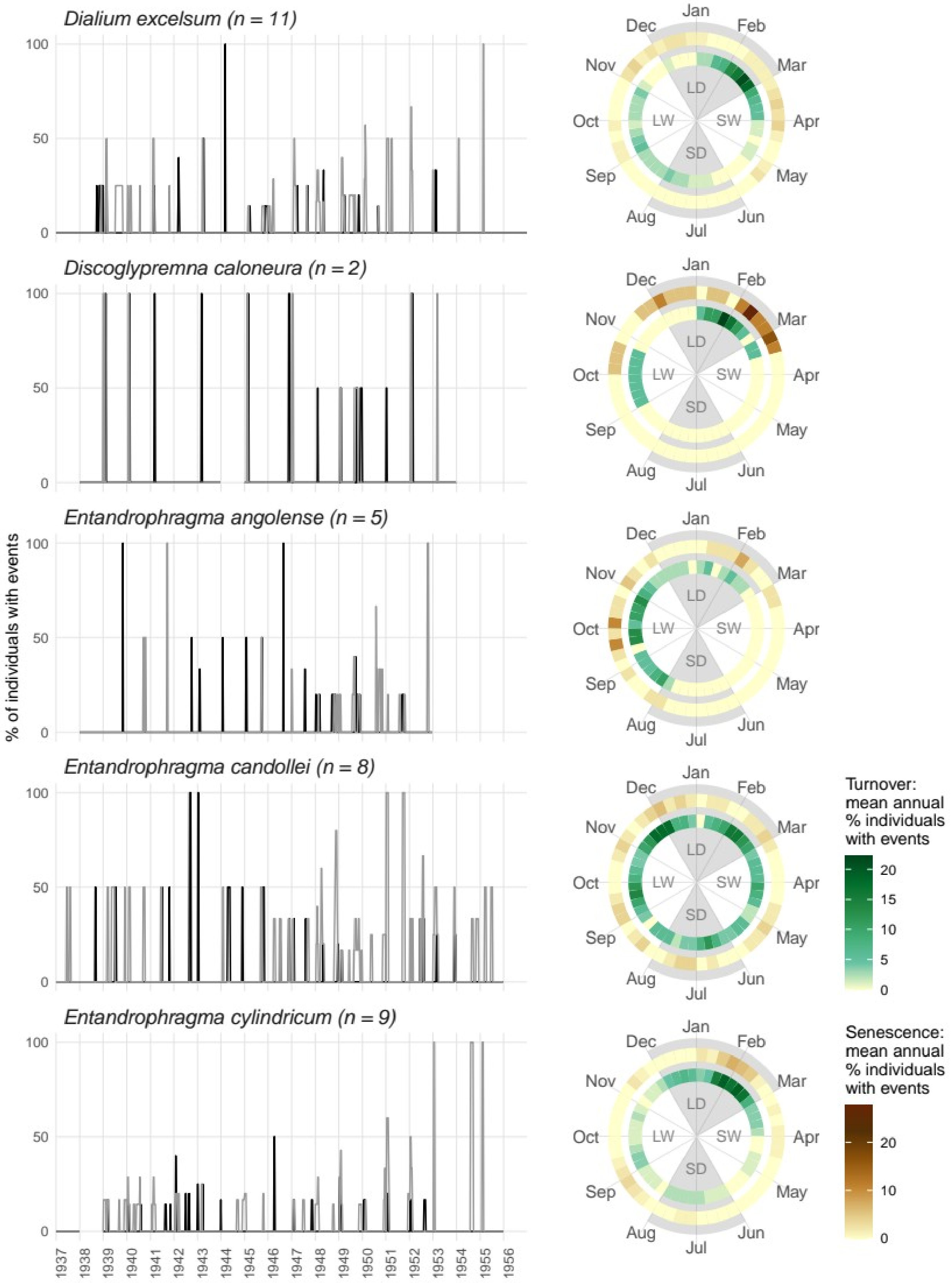

(b) Deciduous – continued

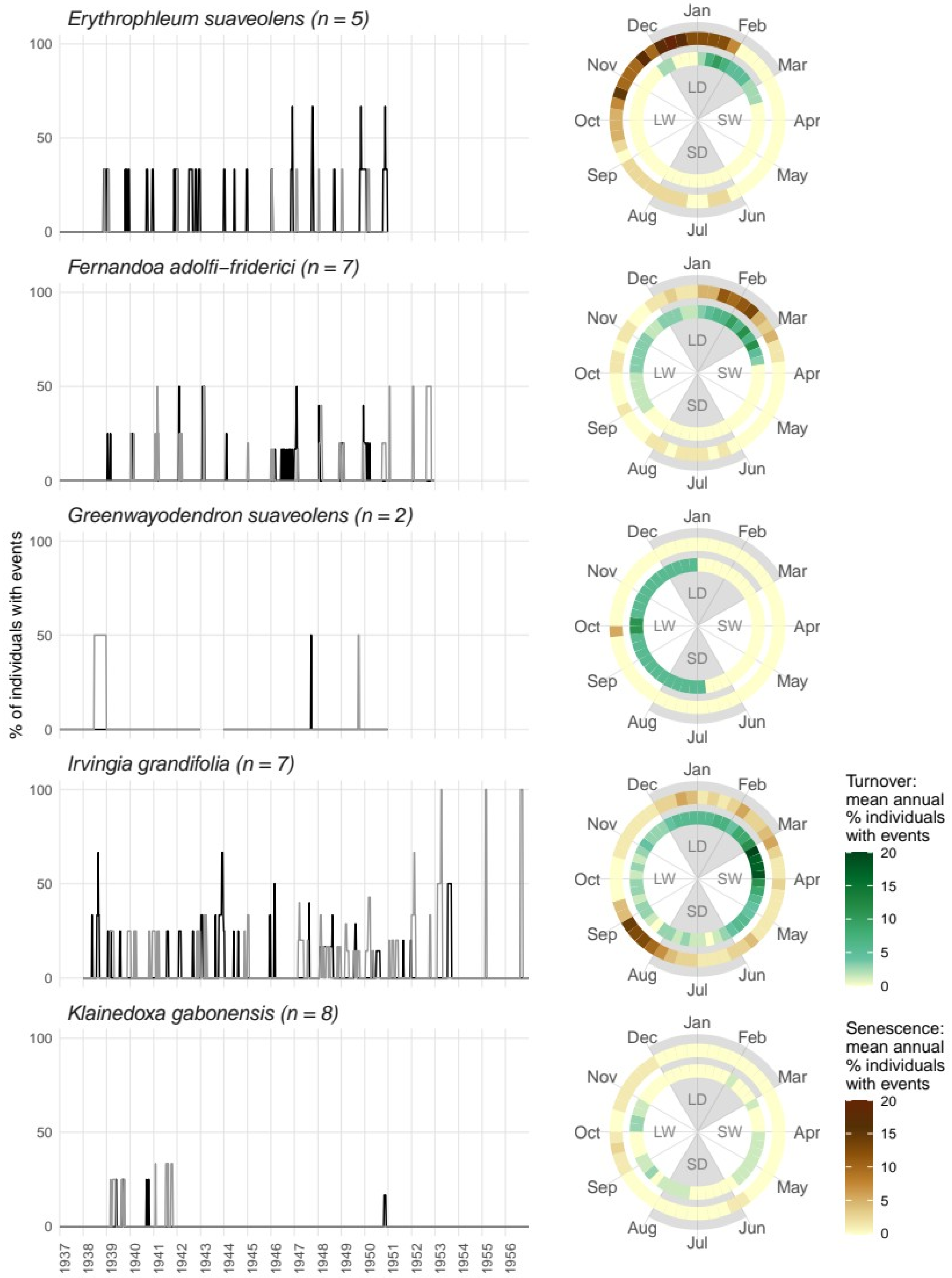

(b) Deciduous – continued

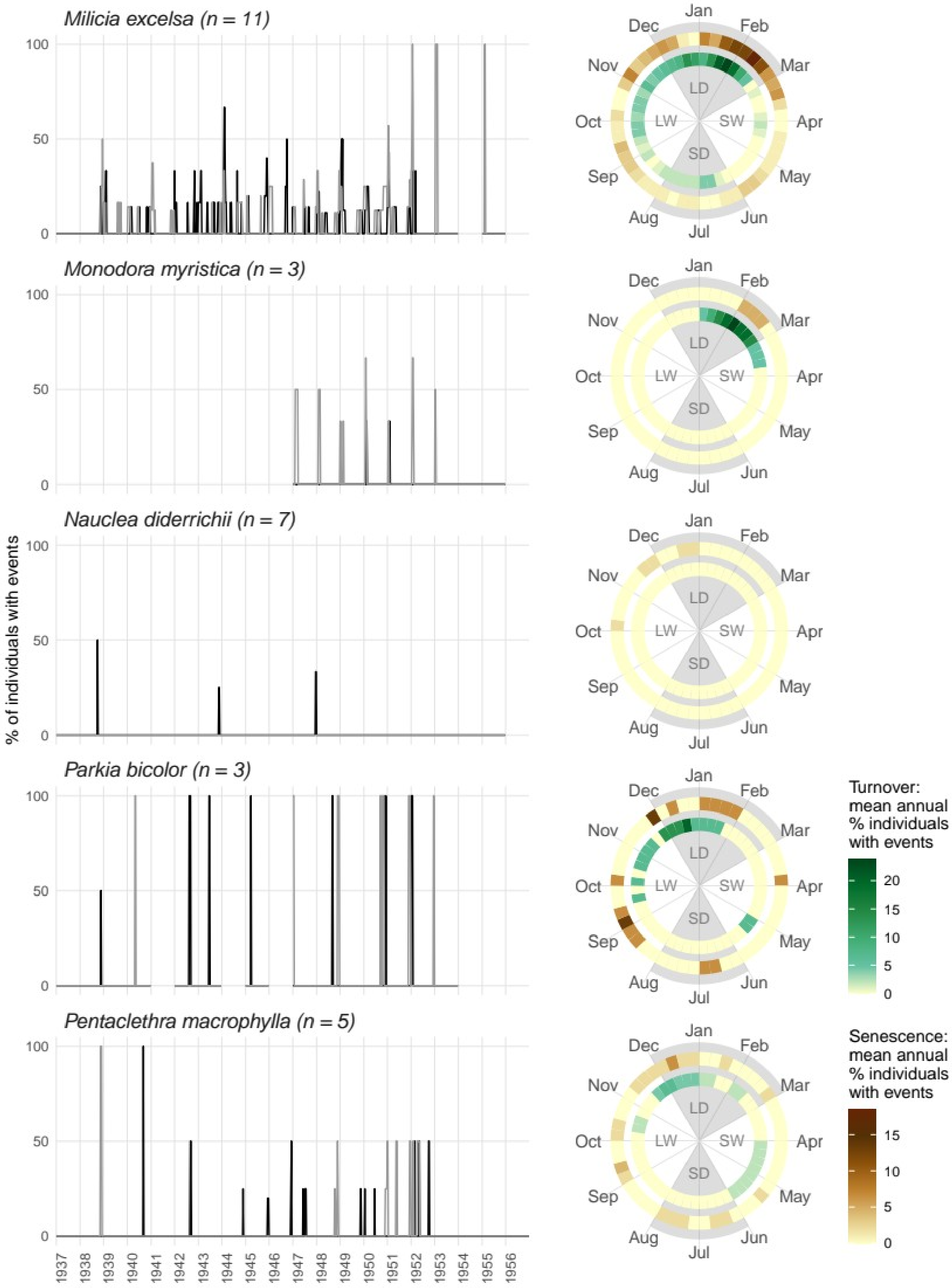

(b) Deciduous – continued

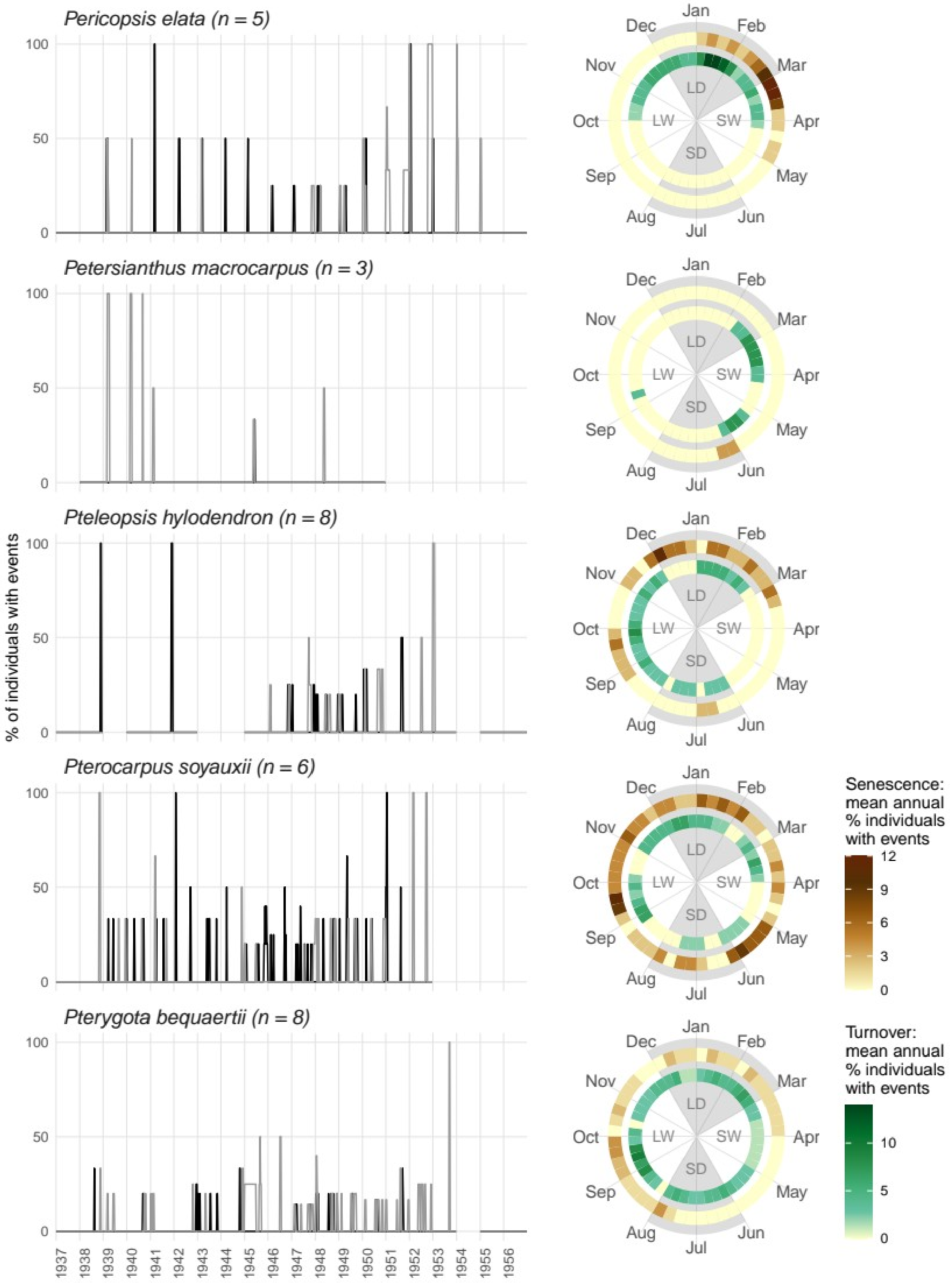

(b) Deciduous – continued

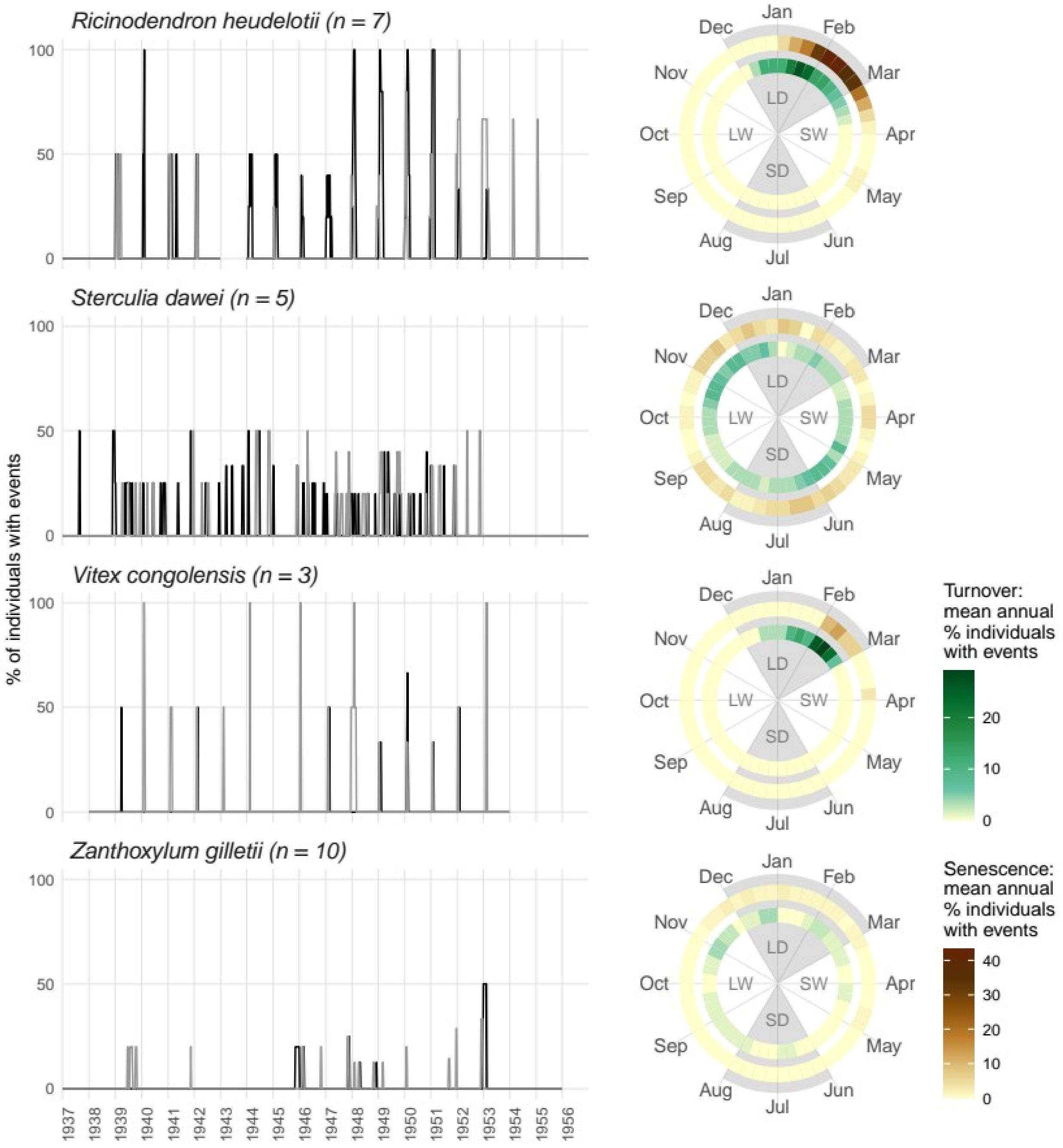

(b) Deciduous – continued

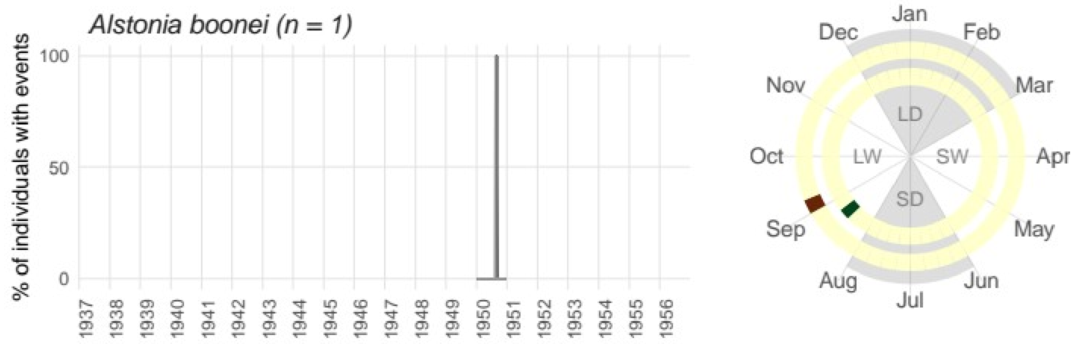

(c) Unclassified

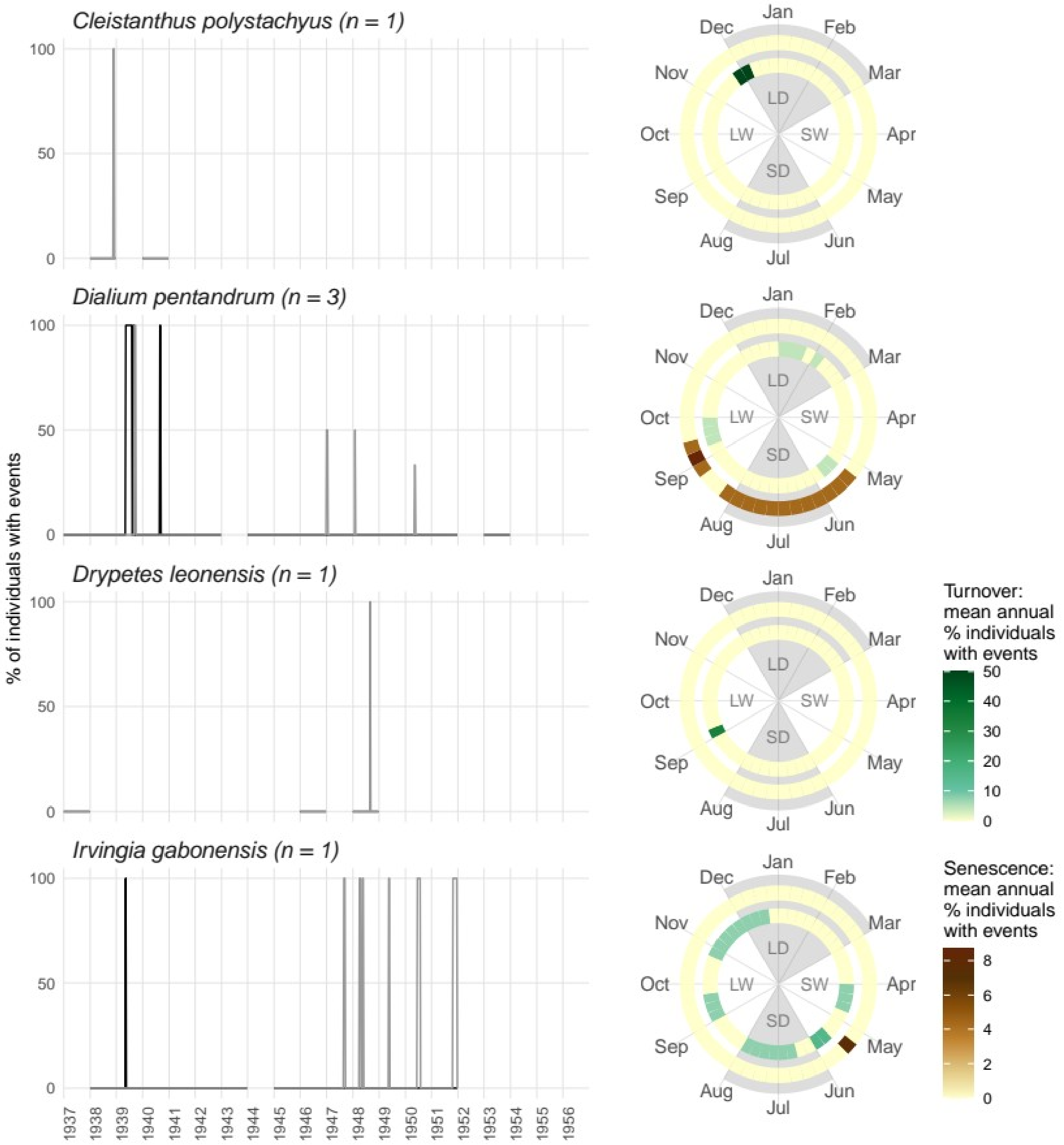

(c) Unclassified – continued

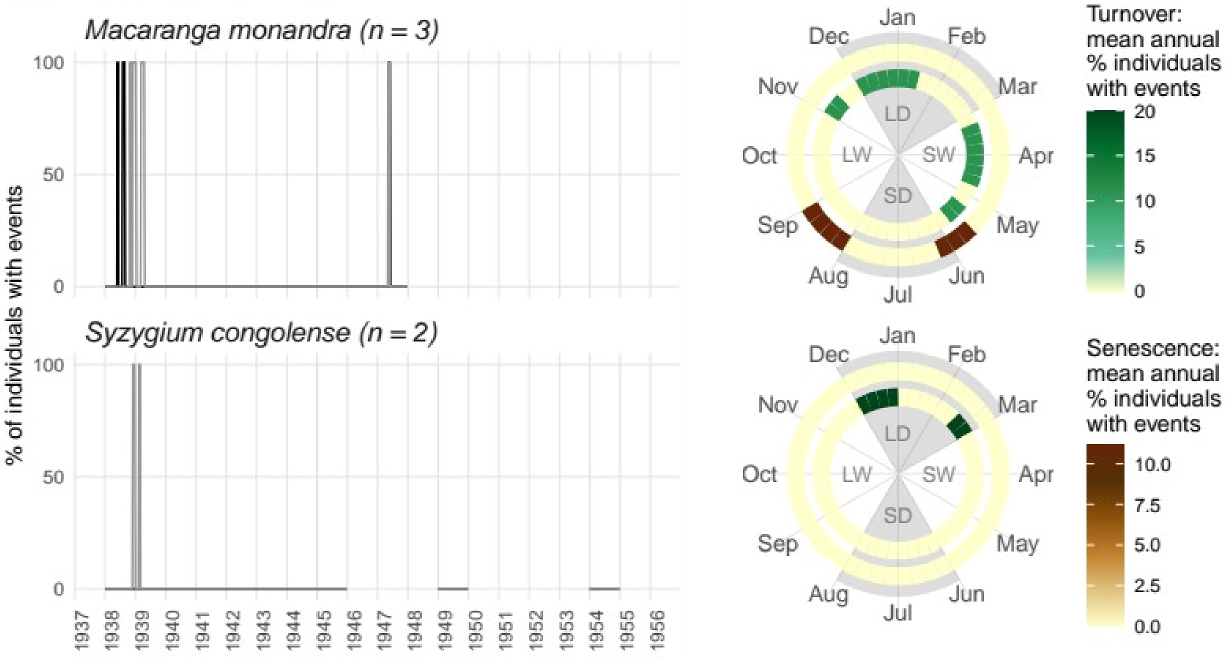

Supplement: Supplementary file 1 — Data S1: [file PEI3-5-e10136-s001.pdf]
